# Epigenetic silencing of *Oct4* by a complex containing SUV39H1 and *Oct4* pseudogene lncRNA

**DOI:** 10.1038/ncomms8631

**Published:** 2015-07-09

**Authors:** Michele Scarola, Elisa Comisso, Rhena Pascolo, Riccardo Chiaradia, Rosa Maria Marion, Claudio Schneider, Maria A. Blasco, Stefan Schoeftner, Roberta Benetti

**Affiliations:** 1 Cancer Epigenetic Group, Laboratorio Nazionale Consorzio Interuniversitario Biotecnologie (LNCIB), Area Science Park, Padriciano 99, Trieste 34149, Italy; 2 Department of Medical and Biological Sciences, University of Udine; Piazzale Massimiliano Kolbe 1, Udine 33100, Italy; 3 Telomeres and Telomerase Group, Spanish National Cancer Research Centre (CNIO), C/Melchor Fernández Almagro, 3, Madrid 28029, Spain; 4 Department for Life Sciences, University of Trieste, Via Weiss 2, Trieste 34128, Italy

## Abstract

Pseudogene-derived, long non-coding RNAs (lncRNAs) act as epigenetic regulators of gene expression. Here we present a panel of new mouse *Oct4* pseudogenes and demonstrate that the X-linked *Oct4* pseudogene *Oct4P4* critically impacts mouse embryonic stem cells (mESCs) self-renewal. Sense *Oct4P4* transcription produces a spliced, nuclear-restricted lncRNA that is efficiently upregulated during mESC differentiation. *Oct4P4* lncRNA forms a complex with the SUV39H1 HMTase to direct the imposition of H3K9me3 and HP1α to the promoter of the ancestral *Oct4* gene, located on chromosome 17, leading to gene silencing and reduced mESC self-renewal. Targeting *Oct4P4* expression in primary mouse embryonic fibroblasts causes the re-acquisition of self-renewing features of mESC. We demonstrate that *Oct4P4* lncRNA plays an important role in inducing and maintaining silencing of the ancestral *Oct4* gene in differentiating mESCs. Our data introduces a sense pseudogene–lncRNA-based mechanism of epigenetic gene regulation that controls the cross-talk between pseudogenes and their ancestral genes.

## Abstract (web summary)

Pseudogene derived long non-coding RNAs (lncRNAs) can regulate the expression of their ancestral genes. Here, the authors show that the *Oct4* pseudogene *OctP4* lncRNA plays an important role in inducing and maintaining silencing of the ancestral Oct4 gene in differentiating mouse embryonic stem cells.

## Graphical abstract

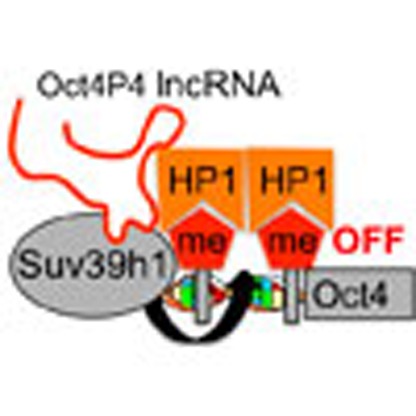

Oct4 is a member of the POU family of transcription factors that controls early embryonic differentiation and is essential to maintain mESC pluripotency and self-renewal^1^,^2^,^3^,^4^,^5^,^6^. *Oct4* is expressed in germ cells, the inner cell mass of pre-implantation embryos, embryonic stem cells (ESC) but also in different types of human cancer^7^,^8^,^9^,^10^. *Oct4* is essential to establish the core transcriptional network maintaining pluripotency and self-renewal in mESCs^1^,^2^,^3^,^4^. Importantly, ectopic expression of the key mESC self-renewal transcription factors *Oct4*, *Sox2*, *Klf4* and *c-Myc* induces pluripotency in differentiated cells, resulting in induced pluripotent stem (iPS) cells^11^. Out of this basic set of transcription factors, *Oct4* is the only indispensible factor for iPS cell generation^11^,^12^. This highlights a central role of *Oct4* for ESC pluripotency and self-renewal. Given the key role of *Oct4* in mESC self-renewal, discovering pathways that control *Oct4* expression has primary importance. Induction of ESC differentiation leads to rapid transcriptional and epigenetic silencing of *Oct4* (refs 13, 14, 15, 16, 17, 18). Other pathways that regulate *Oct4* expression involve post-translational modifications or miRNAs^19^,^20^,^21^,^22^,^23^,^24^,^25^. A growing body of evidence indicates an important role of pseudogene-derived RNAs in regulating the expression of their ancestral gene, either by modulating chromatin structure via anti-sense transcription, short interfering RNA (siRNA) formation or sponging miRNAs from their ancestral genes by acting as competing endogenous RNAs (ceRNAs)^26^,^27^,^28^,^29^,^30^,^31^,^32^. Recent studies demonstrate the existence of various human *OCT4* pseudogenes and anticipate a relevance for pseudogene-derived ncRNAs in the regulation of the ancestral *OCT4* gene in ESCs but also cancer^33^,^34^,^35^,^36^,^37^,^38^,^39^,^40^,^41^,^42^,^43^,^44^. To this end, the human *OCT4* pseudogene *OCT4-pg4* was demonstrated to sponge *OCT4*-specific miRNAs in hepatocellular carcinoma cells^45^; human *OCT4-pg5* anti-sense transcripts reduce *OCT4* promoter activity and mouse *OCT4-pg1* impacts on mesenchymal stem cell differentiation via a still unknown mechanism^39^,^46^.

However, the role of *Oct4* pseuodogenes in controlling mESC pluripotency and self-renewal remains unknown.

Here we identify a panel of novel, processed murine *Oct4* pseudogenes and report on a *trans*-epigenetic cross-talk between an X-linked *Oct4* pseudogene and the ancestral Oct4 gene, located on chromosome 17. We found that a long, non-coding RNA transcribed in sense orientation from *Oct4* pseudogene 4 (*Oct4P4*) recruits the H3K9-specific HMTase SUV39H1 to induce epigenetic repression of the promoter of the ancestral *Oct4* gene in *trans*. This mechanism reduces the expression of key self-renewal transcription factors and promotes mESC differentiation. Importantly, functional disruption of the *Oct4P4*–SUV39H1 silencing complex leads to a re-acquisition of self-renewal features, such as *Oct4* expression and the re-activation of telomerase-dependent telomere maintenance mechanisms. This indicates that *Oct4P4* is essential to prevent the re-activation of self-renewal circuits in differentiated cells.

## Results

### *Oct4* pseudogenes expression during mESC differentiation

**Figure 1:**
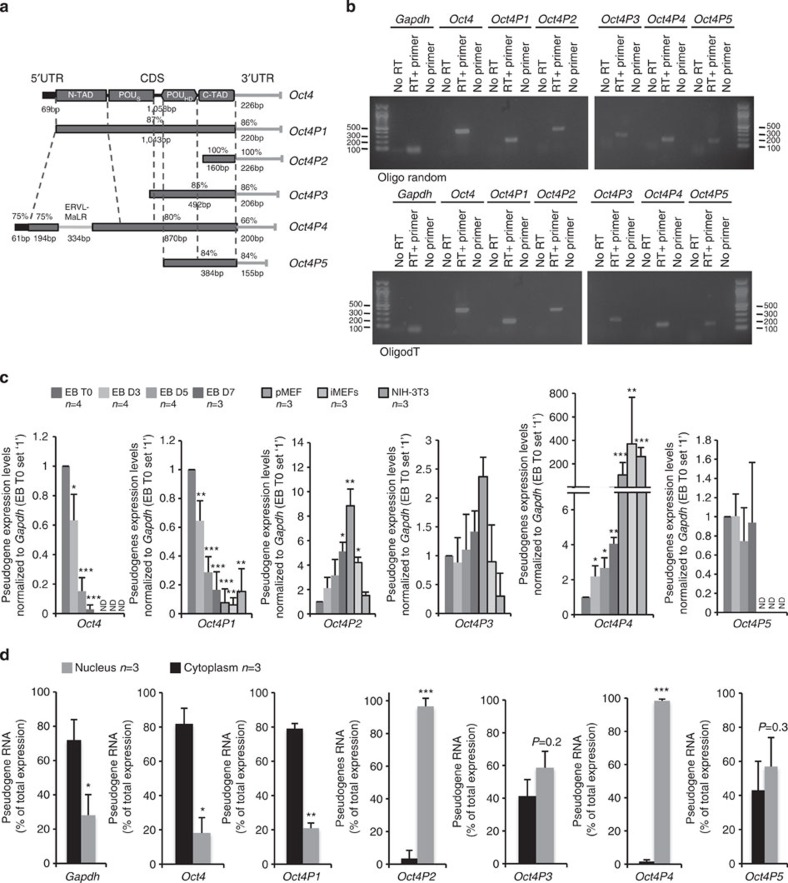
Characterization of murine *Oct4* pseudogenes. (**a**) Schematic representation of *Oct4* and *Oct4* pseudogenes transcripts. Pseudogenes length and percentage of sequence homology to Oct4 are indicated. Regions with high homology to the *Oct4* 5′UTR or 3′UTR are indicated by black boxes or grey lines, respectively. ERVL-MaLRs, spliced region of *Oct4P4* with homology to the LTR of ERVL-MaLRs retrotransposons. OCT4 protein domains are indicated: N-terminal trans-activating domain (N-TAD), POU-specific DNA-binding domain (POU_s_), DNA-binding homeodomain (POU_HD_), C-terminal trans-activating domain (C-TAD). (**b**) Verification of *Oct4* pseudogene expression in mouse embryonic stem cells (mESCs) by semi-quantitative PCR using random primed (top panel) or oligo-dT primed (bottom panel) cDNA. RT+primer, standard cDNA synthesis; no primer, cDNA synthesis without primers; no RT, cDNA synthesis without reverse transcriptase. PCR products are visualized on a 1.2% agarose gel. (**c**) *Oct4* lncRNA pseudogenes expression in self-renewing mESCs (EB T0) and embryoid body (EB) aggregates at day 3 (EB D3), day 5 (EB D5) and day 7 (EB D7); pMEFs; immortalized pMEFs and NIH-3T3 fibroblasts. *Oct4* pseudogenes expression was normalized to *gapdh*. ND, not detectable. (**d**) Subcellular localization of *Oct4* pseudogene lncRNA in self-renewing mESCs. Expression values are shown as percentage of total RNA expression. *n*, number of independent experiments; error bars indicate s.d.; a Student's t-test was used to calculate statistical significance values: *P<0.05; **P<0.01; ***P<0.001.

Recent studies report the existence of eight *OCT4* pseudogenes in the human and one *Oct4* pseudogene (*Oct4-pg1*, here called ‘*Oct4P1*') in the mouse genome^36^,^38^,^39^,^40^. Performing genome blast analyses using the mouse *Oct4 transcript 1* (*Pou5f1*) complementary DNA (cDNA) as reference sequence, we identified four additional candidate pseudogenes (*Oct4P2, Oct4P3, Oct4P4* and *Oct4P5*) that show high homology to portions of the open reading frame and untranslated regions (UTRs) of the ancestral *Oct4* transcript *1* (*Pou5f1*) (Fig. 1a; Supplementary Fig. 1a). Using primers specific for each Oct4 pseudogene, we were able to PCR amplify all candidate pseudogenes from random or oligo-dT primed cDNA prepared from mESC total RNA (Fig. 1b). No PCR product was obtained when cDNA synthesis was carried out in the absence of primers or reverse transcriptase, thus excluding RNA self-priming or contamination of PCR reactions with genomic DNA. The identity of all *Oct4* pseudogene-derived RNAs was sequence validated (Supplementary Fig. 1b). We conclude that *Oct4P2, Oct4P3, Oct4P4* and *Oct4P5* represent novel, processed *Oct4* pseudogenes that are expressed in mESCs. Interestingly, Oct4P4 shows homology to the entire mature *Oct4* mRNA but has acquired a 334 bp insertion that shows high homology to the LTR element of the ERVL-MaLRs retrotransposon family (Fig. 1a). Remarkably, this insert is subjected to RNA splicing (Supplementary Fig. 1c). To test whether *Oct4* pseudogenes are subjected to transcriptional regulation, we prepared total RNA from self-renewing mESC (EB T0) and mESCs that were subjected to embryoid body (EB) *in vitro* differentiation for 3 (EB D3), 5 (EB D5) or 7 (EB D7) days (see Supplementary information). As expected, the ancestral *Oct4* gene is subjected to efficient transcriptional repression during 7 days of EB differentiation (Fig. 1c). We found that EB differentiation is paralleled by a significant reduction of *Oct4P1* and activation of *Oct4P2* and *Oct4P4* expression. EB differentiation does not significantly impact on *Oct4P3* and *Oct4P5* expression (Fig. 1c). Remarkably, *Oct4P4* long non-coding RNA (lncRNA) levels are 150–300-fold higher in primary mouse embryonic fibroblasts (pMEFs) or immortalized mouse embryonic fibroblast compared with undifferentiated mESCs (Fig. 1c). Considering the lower RNA content of mESC compared with pMEFs^47^, we found that *Oct4P4* lncRNA levels are more than 600-fold higher in pMEFs compared with mESCs (Supplementary Fig. 1e). Information on subcellular localization of lncRNAs can provide important information about a possible function as nuclear-restricted epigenetic regulator or cytoplasmatic ceRNA^27^,^48^. Pseudogene-specific quantitative reverse transcription–PCR (RT–PCR) using total RNA from nuclear and cytoplasmatic fractions of undifferentiated mESCs revealed that *Oct4P2* and *Oct4P4* are predominantly located in the nucleus (Fig. 1d). *Oct4P1*, *gapdh* and the ancestral *Oct4* mRNA show enrichment in the cytoplasm; *Oct4P3* and *Oct4P5* did not show specific enrichment in nucleus or cytoplasm of mESCs (Fig. 1d). Together, these data indicate that a subset of RNAs derived from Oct4 pseudogenes show controlled intracellular localization and tight transcriptional regulation, anticipating a potential role in the control of mESC self-renewal. Unique properties of the *Oct4P4* lncRNA such as extended homology to the ancestral Oct4 gene, nuclear localization, splicing, efficient upregulation during mESC differentiation and the virtual absence of relevant ORFs (Supplementary Fig. 1d) prompted us to investigate a possible role of *Oct4P4* in the control of mESC differentiation and self-renewal.

### *Oct4P4* lncRNA represses the expression of ancestral *Oct4*

**Figure 2:**
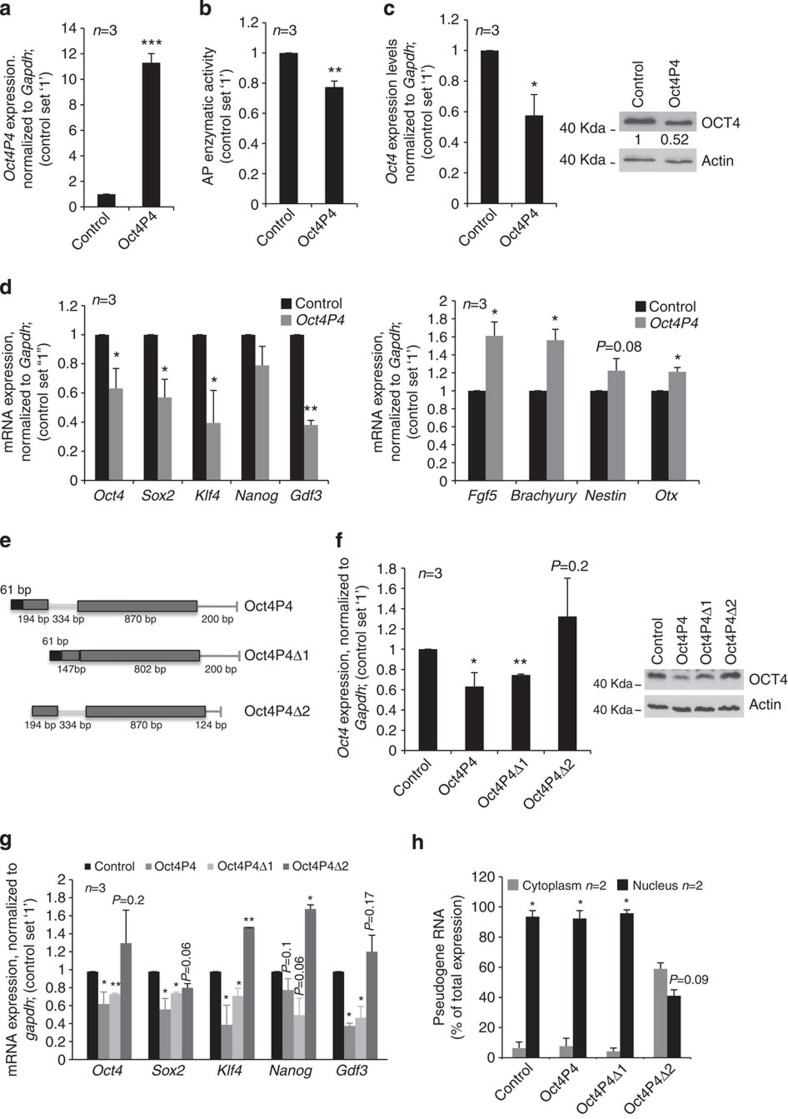
***Oct4P4*** **impairs mESC self-renewal potential.** (**a**) Quantitative real-time PCR determining *Oct4P4* lncRNA expression levels in mESCs stably overexpressing *Oct4P4*. *Oct4P4* expression was normalized to *gapdh*. (**b**) Spectrophotometric measurement of alkaline phosphatase (AP) activity in mESC stably overexpressing *Oct4P4*. (**c**) Ectopic *Oct4P4* expression in self-renewing mESC reduces the expression of *Oct4* as determined by quantitative real-time PCR (left panel) or OCT4 western blotting (right panel). Expression values were normalized against *gapdh*; ACTIN was used as a loading control in western blotting experiments. Numbers represent OCT4/ACTIN ratios (**d**) Quantitative real-time PCR analysis of self-renewal marker genes (left panel) or markers of early mESC differentiation (right panel) in mESCs stably overexpressing *Oct4P4*. Expression levels were normalized against *Gapdh*. (**e**) Schematic representation of full-length and *Oct4P4* deletion constructs. Oct4P4Δ1 lacks intronic ERVL-MaLR homology region and flanking sequences. Oct4P4Δ2 carries a 60 nt and 76 nt deletion at the 5′ and 3′ end of full-length *Oct4P4*, respectively. (**f**) Quantitative real-time PCR analysis using mESCs stably overexpressing *Oct4P4*, *Oct4P4Δ1* and *Oct4P4Δ2*. Expression values were normalized against *gapdh* (left panel). OCT4 western blotting analysis in mESCs stably overexpressing *Oct4P4, Oct4P4Δ1* or *Oct4P4Δ2*. ACTIN was used as a loading control (right panel). *Oct4P4Δ2* does not alter Oct4 expression. (**g**) Expression of pluripotency marker genes in mESCs ectopically expressing *Oct4P4*, *Oct4P4Δ1* or *Oct4P4Δ2*, as determined by quantitative real-time PCR analysis. Expression values were normalized against *gapdh*. (**h**) Subcellular localization of *Oct4P4*, *Oct4P4Δ1* and *Oct4P4Δ2* in mESCs stably expressing the indicated construct, as determined by real-time PCR. Expression values are shown as percentage of total expression. *n*, number of independent experiments carried out; error bars indicate s.d.; ND; not detected; a Student's *t*-test was used to calculate statistical significance values: *P<0.05; **P<0.01; ***P<0.001.

Next, we were interested in testing whether the existence of *Oct4P4* sense and anti-sense transcripts could provide a possible inroad in understanding the role of Oct4P4 in mESC. Performing sense/anti-sense-specific RT–PCR, we were not able to detect anti-sense transcripts originating from the *Oct4* and *Oct4P4* genes. We therefore concluded that a putative function of *Oct4P4* in mESCs must arise from *Oct4P4* sense transcripts (Supplementary Fig. 2a). Investigating a possible role for *Oct4P4* in mESC biology, we stably overexpressed sense *Oct4P4* lncRNA in self-renewing mESCs. We found that stable overexpression of *Oct4P4* resulted in a 23% reduction of alkaline phosphatase activity, a classic marker of mESC self-renewal potential (Fig. 2a,b). Remarkably, *Oct4P4* overexpression reduced AP activity to a similar extent like RNA interference (RNAi)-mediated depletion of endogenous *Oct4* (Supplementary Fig. 2b,c). Importantly, *Oct4P4* overexpression caused a 50% reduction of OCT4 protein levels (Fig. 2c). Reduced alkaline phosphatase activity and OCT4 protein levels were found to be paralleled by significantly reduced mRNA expression of key pluripotency transcription factors such as *Oct4*, *Sox2*, *Klf4* and *Gdf3* and increased expression of early mESC differentiation markers such as *Fgf5*, *Brachyury*, *Nestin* and *Otx2* (Fig. 2d). Together, this indicates that ectopic expression of *Oct4P4* impairs mESC self-renewal by interfering with transcriptional regulatory circuits that control pluripotency.

To identify functionally relevant lncRNA domains, we created a panel of *Oct4P4* expression vectors lacking *Oct4P4* sequence elements with lowest sequence homology to the ancestral *Oct4* cDNA (Fig. 2e). We found that stable overexpression of Oct4P4Δ1 that lacks a 449 nucleotide region comprising the 334 bp ERVL-MaLRs retrotransposon homology sequence was still able to reduce *Oct4* mRNA and protein levels, recapitulating the effect of full-length Oct4P4 (Fig. 2f). In contrast, overexpression of Oct4P4Δ2, lacking sequences with high homology to the *Oct4* 5′ and 3′ UTR regions were not able to reduce *Oct4* expression in mESCs (Fig. 2f). Oct4P4Δ1 overexpression significantly reduces the expression of pluripotency transcription factors. In contrast, Oct4P4Δ2 overexpression does not impact on self-renewing transcriptional circuits in self-renewing mESCs (Fig. 2g). These data suggest that *Oct4P4* 5′ and 3′UTR portions are important to reduce *Oct4* expression. We therefore created additional *Oct4P4* deletion constructs centred on regions located at the 5′ and 3′ termini of *Oct4P4* lncRNA. Deleting the *Oct4P4*-5′UTR (Oct4P4Δ5′UTR) or *Oct4P4*-3′UTR (Oct4P4Δ3′UTR) resulted a reduced efficiency of *Oct4* repression when compared with overexpression of full-length *Oct4P4* lncRNA (Supplementary Fig. 3a,b). Ectopic expression of the 5′UTR or 3′UTR homology elements of the *Oct4P4* lncRNA (Oct4P4-5′UTR or Oct4P4-3′UTR, respectively) did not impact on the expression of the ancestral *Oct4* gene (Supplementary Fig. 3c,d). Together these data indicate that the 5′UTR and 3′UTR homology region of *Oct4P4* collaborate to repress the expression of the ancestral *Oct4* gene. Subcellular fractionation experiments revealed that ectopic *Oct4P4*Δ*1* RNA maintains nuclear localization (Fig. 2h). In contrast, deletion of both, 5′ and 3′ UTR homology regions resulted in a non-specific subcellular distribution of *Oct4P4*Δ*2* RNA in mESCs (Fig. 2h). Finally, individually deleting the 5′UTR or 3′UTR homology regions of *Oct4P4* caused a reduced enrichment of Oct4P4Δ5′UTR or Oct4P4Δ3′UTR RNAs in the nucleus (Supplementary Fig. 3e). This indicates that the 5′UTR or 3′UTR are important to mediate nuclear localization of *Oct4P4*. Of note, overexpression of *Oct4P4* did not cause a subcellular de-localization of ancestral *Oct4* or *gapdh* mRNAs, excluding that *Oct4P4* impairs mESC self-renewal by de-localizing the ancestral *Oct4* mRNA (Supplementary Fig. 3f). We conclude that the *Oct4P4* pseudogene gives rise to a sense lncRNA that employs nuclear-restricted molecular mechanisms to reduce the expression of pluripotency transcription factors, finally leading to impaired mESC self-renewal.

### *Oct4P4* lncRNA orchestrates silencing of the *Oct4* promoter

**Figure 3:**
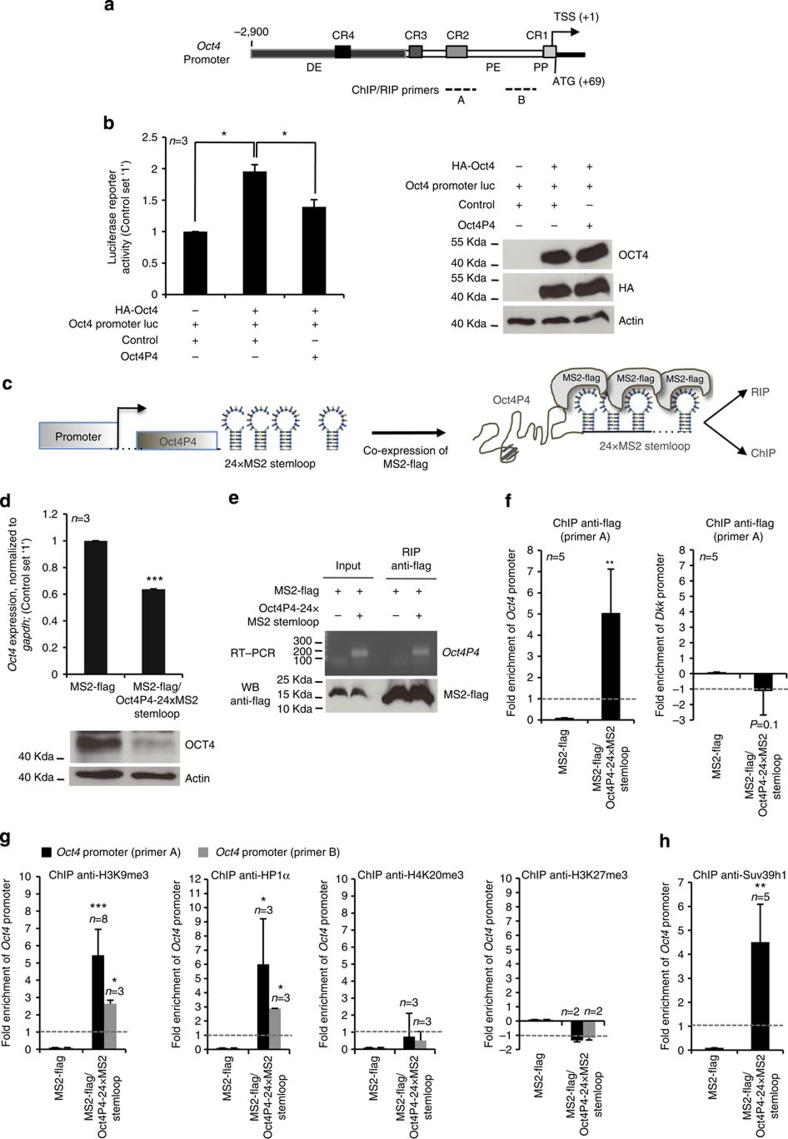
***Oct4P4*** **mediates the imposition of H3K9me3 and HP1α at the** ***Oct4*** **gene.** (**a**) Schematic representation of the mouse *Oct4* promoter region showing the location of the specific distal enhancer (DE), proximal enhancer (PE), proximal promoter (PP) sites and the conserved promoter/enhancer regions (CR1, CR2). The locations of PCR amplicons (A and B) used in ChIP experiments are indicated. (**b**) Luciferase reporter assay demonstrating that *Oct4P4* controls *Oct4* promoter activity. NIH-3T3 cells were co-transfected with indicated vectors. Luciferase activity was assayed 72 h after co-transfection (left panel); western blotting experiments using the indicated antibodies were performed in parallel (right panel). (**c**) Schematic representation of the *Oct4P4-24 × MS2* stem loop construct used to generate mESCs stably expressing MS2-flag and *Oct4P4-24 × MS2* stem loop RNA. (**d**) *Oct4P4-24 × MS2* stem loop RNA overexpression in mESC reduces the expression of ancestral *Oct4*, as determined by quantitative RT–PCR; expression values were normalized against *Gapdh* (top panel); Result was validated by OCT4 western blotting, using ACTIN as a loading control (bottom panel). (**e**) RIP using mESCs overexpressing MS2-flag/*Oct4P4-24 × MS2* stem loop RNA or MS2-flag control cells. Agarose gel electrophoresis after semi-quantitative PCR demonstrates the presence of *Oct4P4-24 × MS2* stem loop RNA in anti-flag RIP experiments (top panel). Immunoprecipitation of MS2-flag was validated by western blotting (bottom panel) (**f**) ChIP using specific anti-flag antibodies followed by quantitative RT–PCR using lysates from MS2-flag/*Oct4P4-24 × MS2* stem loop or MS2-flag control mESCs. MS2-flag is enriched at the *Oct4* promoter in *Oct4P4-24 × MS2* stem loop mESCs (left panel); no enrichment was detected at the unrelated *Dkk* promoter (right panel). ChIP data were quantified versus input and unrelated HA-specific antibodies. (**g**) ChIP analysis of *Oct4* promoter region in mESCs stably overexpressing *Oct4P4* using indicated antibodies. Quantitative RT–PCR was performed to measure promoter enrichment. *Oct4P4* overexpression drives H3K9me3 and HP1α enrichment at Oct4 promoter region. (**h**) ChIP analysis of *Oct4* promoter region in MS2-flag/*Oct4P4-24 × MS2* stem loop and control MS2-flag mESCs using SUV39H1-specific antibodies. *Oct4P4-24 × MS2* stem loop results in an enrichment of SUV39H1 at the *Oct4* promoter. *n*, number of independent experiments carried out; WB, western blotting; error bars indicate s.d.; a Student's *t*-test was used to calculate statistical significance values: *P<0.05; **P<0.01; ***P<0.001.

Based on the fact that nuclear localization is essential for *Oct4P4* lncRNA function, we speculated that *Oct4P4* might directly interact with the promoter of the ancestral Oct4 gene. To test this hypothesis, we performed *Oct4* promoter luciferase reporter experiments in NIH-3T3 cells that lack expression of endogenous *Oct4* (Fig. 1c, Fig. 3a). Co-transfection of the reporter with a plasmid-encoding haemagglutanin (HA)-epitope-tagged murine OCT4 under the control of the CMV promoter was able to increase Oct4 promoter luciferase reporter activity (Fig. 3b, left panel). Importantly, *Oct4P4* overexpression significantly attenuated this effect without affecting expression levels of ectopically expressed OCT4 protein (Fig. 3b, right panel). This suggests that *Oct4P4*-derived lncRNAs specifically reduced the activity of the Oct4 promoter used in the reporter construct. To provide evidence for a cross-talk between *Oct4P4* ncRNA and the promoter of the ancestral *Oct4* gene in *trans*, we established an ‘Oct4P4-MS2 RNA stem loop' tethering system in mESC^49^. We generated mESCs stably co-expressing a flag-tagged version of MS2 phage coat protein (MS2-flag), as well as *Oct4P4* lncRNA fused to 24 repeats of the MS2 RNA stem loop (24 × MS2 stem loop), that binds with high affinity to MS2 phage coat protein (Fig. 3c; Supplementary Material and Methods). This model system permits the use of anti-flag antibodies to detect *Oct4P4-24 × MS2* stem loop RNA at gene loci by chromatin immunoprecipitation (ChIP) or to identify *Oct4P4* interacting proteins by RNA immunoprecipitation (RIP) (Fig. 3c). mESCs exclusively overexpressing MS2-flag were used as a control cells. In line with previous data, overexpression of MS2 stem loop-tagged *Oct4P4* (*Oct4P4-24 × MS2* stem loop) results in reduced OCT4 protein and mRNA expression (Fig. 3d). Importantly, we were able to efficiently amplify *Oct4P4-24 × MS2* stem loop RNA by RT–PCR in anti-flag RIP experiments using mESCs stably co-expressing MS2-flag and Oct4P4-24 × MS2 stem loop constructs. This demonstrates that MS2-flag forms a complex with the *Oct4P4-24 × MS2* stem loop RNA *in vivo* (Fig. 3e). To demonstrate that *Oct4P4-24 × MS2* stem loop RNA localizes to the endogenous *Oct4* promoter region in MS2-flag expressing mESCs, we performed ChIP experiments using a set of primer pairs that specifically amplify the promoter of the ancestral *Oct4* gene (Fig. 3a; primer pairs A, B)^50^. Performing ChIP using anti-flag antibodies, we were able to demonstrate the presence of MS2-Flag at the conserved *Oct4* promoter/enhancer region CR2 in MS2-flag/*Oct4P4-24 × MS2* stem loop RNA-expressing mESCs (Fig. 3f). Importantly, MS2-flag control mESCs that lack *Oct4P4-24 × MS2* stem loop RNA expression did not show association of MS2-flag with the *Oct4* promoter (Fig. 3f). Anti-flag ChIP did not immunoprecipitate the promoter region of the unrelated *Dkk* gene in control cells (Fig. 3f, right panel).

Together, our data demonstrate that the *Oct4P4 lncRNA* is required to localize MS2-flag to the *Oct4* promoter in MS2-flag/Oct4P4-24 × MS2 stem loop RNA-expressing mESCs. This data support a model where a sense-oriented *Oct4P4* lncRNA can localize in *trans* to the promoter of the *Oct4* gene.

Nuclear, lncRNAs such as *XIST*, *HOTAIR* or pericentric satellite repeats plays a key role in recruiting epigenetic repressors such as the H3K9-specific SUV39H Histone HMTases or H3K27-specific HMTase containing Polycomb repressive complexes^51^,^52^,^53^. We consequently speculated that upregulation of *Oct4P4* ncRNA expression during mESC differentiation (Fig. 1c) might direct epigenetic silencing complexes to the endogenous *Oct4* promoter. Taking advantage of our *Oct4P4*-MS2 RNA tethering mESC model system, we found that *Oct4P4-24 × MS2* stem loop RNA overexpression in MS2-flag mESCs result in a fivefold increase of H3K9me3 at conserved *Oct4* promoter/enhancer regions (CR1, CR2; detected by primer pairs A and B), as determined by ChIP (Fig. 3a,g). Consistent with the reported high binding affinity of HP1 to H3K9me3 (ref. 54), we found a sixfold increase of HP1α at the *Oct4* promoter in our model mESC line (Fig. 3g). Of note, *Oct4P4-24 × MS2* stem loop RNA overexpression in MS2-flag mESCs does not impact on the abundance of H4K20me3 or H3K27me3 at the *Oct4* promoter (Fig. 3g). In line with increased H3K9me3 levels, we found a fourfold increase of the H3K9-specific SUV39H1 HMTase at the *Oct4* promoter of MS2-flag/*Oct4P4-MS2-24 × MS2* stem loop mESCs (Fig. 3h). To individuate *Oct4P4* lncRNA regions that are important for SUV39H1 interaction and SUV39H1 deposition on the *Oct4* promoter, we performed anti-SUV39H1 ChIP and RIP experiments using mESCs stably overexpressing full-length *Oct4P4, Oct4P4Δ5′UTR*, *Oct4P4Δ3′UTR or Oct4P4Δ2* lncRNAs. Importantly, we found that SUV39H1 deposition on the Oct4 promoter was reduced in *Oct4P4Δ5′UTR* and *Oct4P4Δ3′UTR* cells when compared with full-length *Oct4P4* overexpressing cells (Supplementary Fig. 4a). Deletion of the *Oct4* 5′ and 3′ UTR homology regions in *Oct4P4* reduced efficiency of *Oct4P4* lncRNA mediated repression of *Oct4* (Fig. 2f). In line with this, mESCs overexpressing *Oct4P4* lncRNAs lacking the 5′UTR (*Oct4P4Δ5′UTR*) or 3′UTR (*Oct4P4Δ3′UTR*) homology show reduced SUV39H1 abundance at the Oct4 promoter when compared with cells overexpressing the full-length *Oct4P4* lncRNA (Supplementary Fig. 4a). Finally, overexpression of an *Oct4P4* version lacking both the 3′ and 5′ UTR homology region (Oct4P4Δ2) did not result in the accumulation of SUV39H1 at the *Oct4* promoter. This indicates that the 5′ and 3′UTR homology regions are important to localize SUV39H1 to the Oct4 promoter. Accordingly, Oct4P4Δ2 was no longer able to physically interact with SUV39H1 (Supplementary Fig. 4b), further underlying the importance of 5′ UTR and 3′UTR homology regions for *Oct4P4* function.

Altogether, our data show that the *Oct4P4* lncRNA, expressed in sense orientation from X-linked *Oct4P4* locus, mediates the enrichment of H3K9me3, HP1α and SUV39H1 at the *Oct4* promoter, located on chromosome 17. This *trans-*acting silencing mechanism leads to reduced Oct4 expression that finally results in impaired self-renewal potential of mESCs (Fig. 3d,g,h).

### An *Oct4P4*–SUV39H1 complex targets the *Oct4* promoter

We showed that *Oct4P4* lncRNA overexpression leads to chromatin compaction at the promoter of the ancestral *Oct4* gene. We next wished to test whether (i) *Oct4P4* physically interacts with SUV39H1 and (ii) both players are required to induce epigenetic gene silencing at the promoter of the ancestral *Oct4* gene.

**Figure 4:**
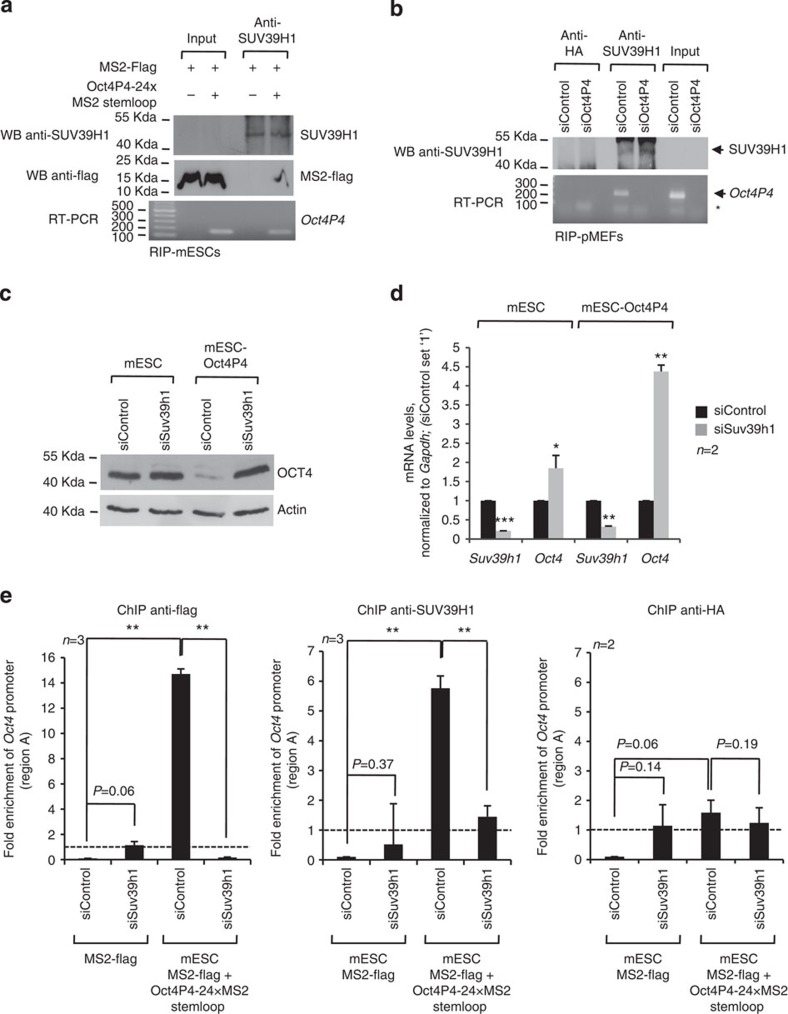
***Oct4P4*** **interacts with SUV39H1 to silence ancestral** ***Oct4*** **in** ***trans***. (**a**) Anti-SUV39H1 RIP using MS2-flag*/Oct4P4-24 × MS2* stem loop mESCs or MS2-flag mESCs cells. Efficiency of anti-SUV39H1 immunoprecipitation was validated by western blotting (top panel). SUV39H1 is not detectable in inputs due to reduced antibody efficiency in western blotting compared with immunoprecipitation (Supplementary Fig. 5a). MS2-flag co-immunoprecipitates with SUV39H1 only in the presence of *Oct4P4-24 × MS2* stem loop RNA, as determined by western blotting (middle panel). Semi-quantitative RT–PCR followed by agarose gel electrophoresis verified the presence of *Oct4P4-24 × MS2-*stem loop RNA in anti-SUV39H1 RIP (bottom panel). (**b**) Anti-SUV39H1 RIP immunoprecipitation of endogenous *Oct4P4* RNA from lysates obtained from pMEFs transfected with control or Suv39h1-specific siRNAs. Efficiency of SUV39H1 immunoprecipitation was validated by western blotting (top panel). *Oct4P4* RNA is undetectable in RIP experiments using lysates from *Suv39h1* depleted cells, demonstrating the specificity of the anti-SUV39H1 antibody (bottom panel). A human influenza haemagglutinin (HA) antibody was used as a control in RIP experiments. (**c**,**d**) *Oct4* expression levels in wild-type (wt) mESCs and mESCs stably overexpressing *Oct4P4* after transient transfected with the indicated siRNA oligos. *Suv39h*1 knockdown increases *Oct4* expression on the protein (**c**) and mRNA level (**d**) as determined by western blotting (**c**) and quantitative real-time PCR (**d**). ACTIN was used as a loading control in western blotting; *Oct4* mRNA levels were normalized to *gapdh*. (**e**) *Oct4* promoter ChIP using MS2-flag control and MS2-flag/*Oct4P4-24 × MS2* stem loop mESCs under *Suv39h1* knock-down conditions. Quantitative real-time PCR on anti-flag immunoprecipitates demonstrates that a *Oct4P4-24 × MS2* stem loop/MS2-flag RNA–protein complex associates with the endogenous *Oct4* promoter only in the presence of *Suv39h1* (left panel). *Suv39h1* knockdown significantly reduces SUV39H1 abundance at the Oct4 promoter, demonstrating specificity of the anti-SUV39H1 antibody (middle panel). An HA antibody was used as a control (right panel). *Dkk* promoter was used as a negative control. *n*, number of independent experiments carried out; WB, western blotting; error bars indicate s.d.; a Student's *t*-test was used to calculate statistical significance values: *P<0.05; **P<0.01; ***P<0.001.

Using MS2-flag/*Oct4P4-24 × MS2* stem loop model mESC, we found that anti-SUV39H1-specific antibodies were able to immunoprecipitate MS2-flag but also *Oct4P4-24 × MS2* stem loop lncRNA (Fig. 4a; Supplementary Fig. 5a). Importantly, also endogenous *Oct4P4* lncRNA was detectable by RT–PCR in anti-SUV39H1 RIP experiments using pMEFs, that are characterized by high *Oct4P4* lncRNA expression levels (Figs 1c and 4b). Of note, *Oct4P4* lncRNA abundance was found to be strongly reduced in anti-SUV39H1 RNA immunoprecipitates after RNAi-mediated reduction of endogenous *Oct4P4*, thus confirming the specificity of our experiments (Fig. 4b).

Next, we wished to validate that SUV39H1 is required to reduce *Oct4* expression by mediating the enrichment of H3K9me3 and its high-affinity binding partner HP1α at the *Oct4* promoter.

RNAi-mediated knockdown of SUV39H1 resulted in significantly increased *Oct4* mRNA and OCT4 protein levels in wild-type mESCs and *Oct4P4* lncRNA-overexpressing mESCs. This provided first evidence that the SUV39H1 HMTase controls *Oct4* expression in mESCs (Fig. 4c,d). To understand the relevance of SUV39H1 for the establishment of a repressive chromatin structure at the endogenous *Oct4* promoter, we performed anti-flag and anti-SUV39H1 ChIP experiments in MS2-flag/*Oct4P4-24 × MS2* stem loop mESCs. As expected, SUV39H1 RNAi resulted in reduced abundance of SUV39H1 and H3K9me3 at the *Oct4* promoter in MS2-flag/*Oct4P4-24 × MS2* stem loop mESCs (Fig. 4e, Supplementary Fig. 5b). Importantly, this effect was paralleled by reduced MS2-flag levels at the *Oct4* promoter, indicative for reduced abundance of *Oct4P4-24 × MS2* stem loop RNA at the ancestral *Oct4* promoter. Together, this indicates that both SUV39H1 and *Oct4P4* lncRNA are required to mediate localization of the silencing complex to the endogenous *Oct4* promoter.

### *Oct4P4* blocks aberrant acquisition of self-renewal features

**Figure 5:**
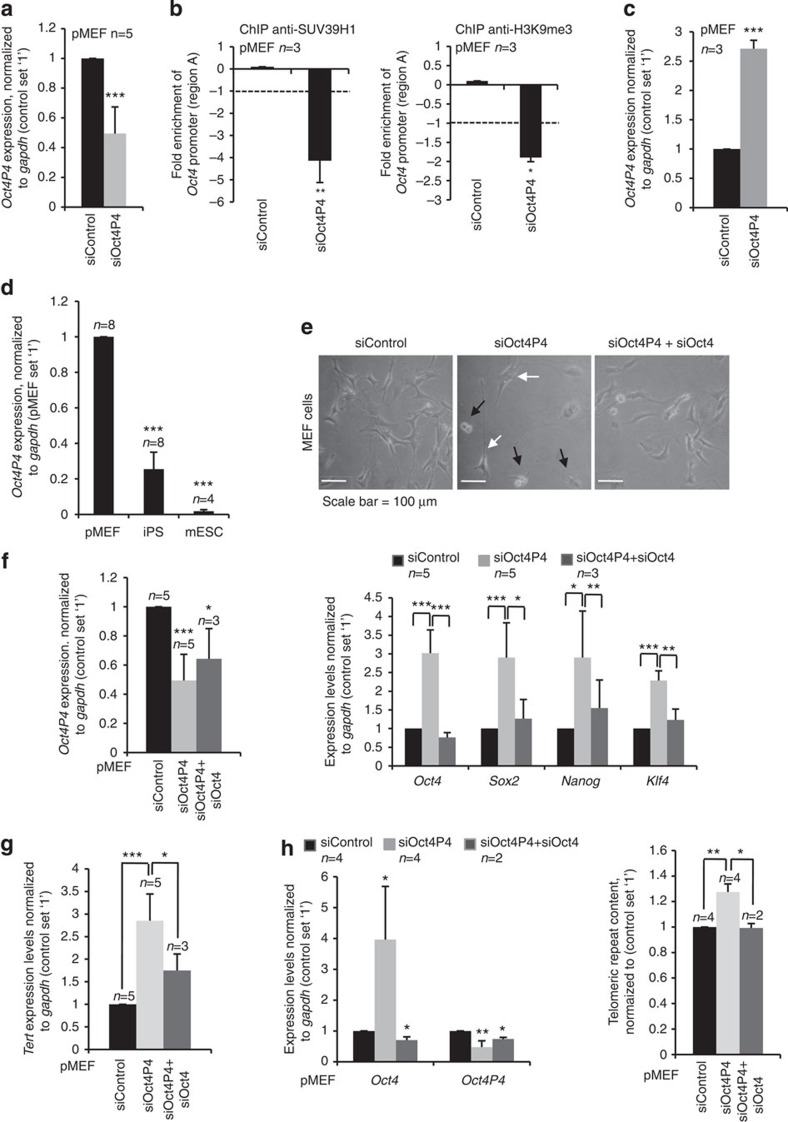
***Oct4P4*** **prevents the activation of features of mESC self-renewal in pMEFs.** (**a**) Quantitative real-time PCR determining *Oct4P4* expression levels in pMEFs transiently transfected with siRNA targeting *Oct4P4*. Control cells were transfected with siRNA control oligos. *Oct4P4* expression was normalized to *gapdh*. (**b**) *Oct4* promoter ChIP analysis using SUV39H1- or H3K9me3-specific antibodies in MEFs transiently transfected with indicated siRNAs. *Oct4P4* siRNA reduces H3K9me3 and SUV39H1 abundance, at the *Oct4* promoter, as determined by quantitative real-time PCR (*Oct4* amplicon A). Dkk promoter was used as a negative control. (**c**) siRNA-mediated knockdown of *Oct4P4* in pMEFs increases *Oct4* expression in quantitative real-time PCR experiments. Expression values were normalized against *gapdh*. (**d**) *Oct4P4* lncRNA expression in pMEFs and their derivative iPS cells. mESCs were included as control. *Oct4P4* expression levels were normalized against *gapdh*. (**e**) Morphology of pMEFs transfected with the indicated siRNAs oligonucleotides. Black arrows indicate cells with altered cell morphology. White arrows indicate pMEFs. Scale bar, 100 μm (**f**) Quantitative real-time PCR analysis of *Oct4P4* lncRNA levels (left panel) and mESC self-renewal marker genes (right panel) in pMEFs transiently transfected with siRNA targeting *Oct4P4* or with a combination of siOct4P4 and siOct4 oligonucleotides. Expression levels were normalized against *gapdh*. Knockdown of *Oct4P4* causes an increased expression of self-renewal marker genes. Reduction of *Oct4* expression in the context of *Oct4P4* knockdown rescues self-renewal marker genes. (**g**) *Tert* mRNA expression level in pMEFs transfected with indicated siRNA oligonucleotides, as determined by quantitative real-time PCR. siRNA-mediated knockdown of *Oct4P4* increases *Tert* expression. Expression levels were normalized against *gapdh*. (**h**) Measurement of telomere repeat content by quantitative real-time PCR. Total genomic DNA from pMEF transiently transfected with indicated siRNA oligonucleotides were analysed using telomere repeat-specific PCR primers (right panel). Alu-equivalent B1 repeats were used as a reference sequence. siRNA-mediated reduction of *Oct4P4* leads to increased telomere repeat content, indicative for increased telomere length. Knock-down efficiency was validated by quantitative RT–PCR (left panel). *n*, the number of independent experiments carried out; error bars indicate s.d.; a Student's *t*-test was used to calculate statistical significance values: *P<0.05; **P<0.01; ***P<0.001.

High *Oct4P4* lncRNA expression levels in pMEFs (Fig. 1c) suggest that the *Oct4P4*–SUV39H1 lncRNA–protein complex has an important role in silencing the endogenous *Oct4* promoter in differentiated cells. In line with this hypothesis, we found that siRNA-mediated knockdown of *Oct4P4* lncRNA in pMEFs results in a fourfold or twofold reduction of SUV39H1 or H3K9me3 at the *Oct4* promoter, respectively (Fig. 5a,b). Importantly, reduced chromatin compaction at the *Oct4* promoter was paralleled by a significant increase in *Oct4* mRNA expression (Fig. 5c). This data was confirmed in experiments using an independent *Oct4P4* lncRNA-specific siRNA oligonucleotide (Supplementary Fig. 6a,b). This indicates that the *Oct4P4*–SUV39H1 complex plays an important role in maintaining silencing of the *Oct4* promoter in pMEFs. Interestingly, we found that *Oct4P4* is efficiently repressed during the conversion of pMEFs to iPS (Fig. 5d). This suggests that *Oct4P4* might represent a barrier to the de-differentiation of pMEFs. To further test this idea, we performed *Oct4P4* loss-of-function experiments in pMEFs. *Oct4P4* knockdown caused a change of the characteristic flat and single-cell morphology of pMEFs to round-shaped cells but also the formation of multi-cellular spheres that grow in suspension or attached on top of pMEFs (Fig. 5e). The formation of spheres or round-shaped cells was inhibited when pMEFs were co-transfected with a mix of siOct4P4 and siOct4 oligonucleotides, indicating that changes in cell morphology are *Oct4* dependent (Fig. 5e,f). Importantly, siOct4P4-induced changes in cell morphology were paralleled by a significantly increased expression of mESC pluripotency transcription factors *Oct4* (threefold), *Sox2* (threefold), *Nanog* (threefold) and *Klf4* (twofold) (Fig. 5f). This data was confirmed in experiments using an independent *Oct4P4*-specific siRNA oligonucleotide (Supplementary Fig. 6c). Remarkably, siOct4P4-dependent increase of pluripotency transcription factor expression was attenuated by contemporary knockdown of *Oct4* (Fig. 5f). Together, this indicates that depletion of *Oct4P4* from pMEFs results in a loss of heterochromatin at the *Oct4* promoter, leading to the re-activation of endogenous Oct4 that promotes the expression of additional key transcription factors of mESC self-renewal. To find evidence that loss of *Oct4P4* activates other biological pathways linked to mESC self-renewal programmes, we studied the impact of *Oct4P4* on telomere maintenance in pMEFs. pMEFs are characterized by progressive telomere shortening due to lack of sufficient telomerase activity. In line with this, re-activation of telomerase activity is a key step for the acquisition of pluripotency during iPS reprogramming of pMEFs^55^. Importantly, we found that 2 consecutive cycles of RNAi-mediated depletion of *Oct4P4* from pMEFs resulted in a 2.6-fold upregulation of the transcript for the catalytic subunit of telomerase, TERT. Importantly, *Tert* mRNA upregulation was paralleled by a concomitant increase in telomeric DNA repeat content, as determined by real-time PCR-based quantification of telomeric DNA repeats in experimental cells (Fig. 5g,h). This data was confirmed in experiments using independent *Oct4P4*-specific siRNA oligonucleotides (Supplementary Fig. 6c,d). This indicates that *Oct4P4* depletion re-activates telomerase expression to drive telomere elongation. Remarkably, these effects were attenuated when pMEFs where transfected with a mix of siRNAs that target *Oct4P4* lncRNA and the ancestral *Oct4* mRNA (Fig. 5g,h).

Together, our data demonstrate that depleting *Oct4P4* from differentiated pMEFs results in the re-activation of basic features of mESC pluripotency, such as self-renewal transcription factor expression and activation of telomerase-dependent mechanisms of telomere maintenance. This further indicates that *Oct4P4* has an important role in preventing the aberrant expression of self-renewal promoting transcription factors in differentiated cells.

## Discussion

**Figure 6:**
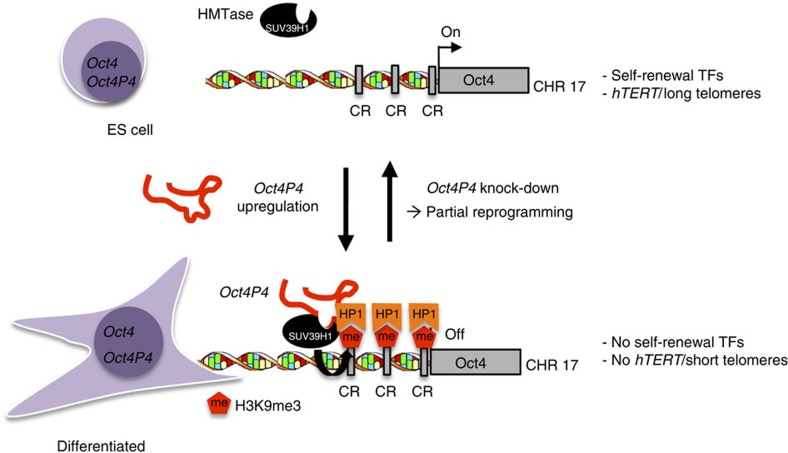
**A model for** ***Oct4P4*** **lncRNA function.** The nuclear, long non-coding *Oct4P4* is upregulated during mESC differentiation and recruits the H3K9-specific HMTase SUV39H1 to impose H3K9me3 and HP1α at the promoter of ancestral *Oct4* gene in *trans*, leading to *Oct4* gene silencing. Depletion of *Oct4P4* lncRNA in differentiated pMEFs results in re-activation of *Oct4* expression and the acquisition of classic features of self-renewal such as increased expression of self-renewal transcription factors and activation of telomerase-dependent telomere maintenance mechanisms. This indicates that *Oct4P4* prevents the aberrant expression of self-renewal programmes in differentiated cells.

Pseudogene-derived transcripts have been reported to impact on the expression of ancestral protein-coding gene using molecular pathways based on RNA sequence homology. Pseudogenes derived transcripts were demonstrated to control ancestral gene by producing endogenous siRNAs, sponging miRNAs by acting as ceRNAs or altering the stability of the ancestral mRNA^28^,^29^,^30^,^31^,^32^,^56^. In addition, anti-sense pseudogene transcripts can impact on the promoter activity of ancestral genes^30^,^36^,^57^,^58^. In this study, we used mESCs to identify a set of novel transcripts derived from putative *Oct4* pseudogenes (*Oct4P1, Oct4P2, Oct4P3 Oct4P4* and *Oct4P5*). Tight regulation of subcellular localization and expression during mESC differentiation anticipate a defined role for a subset of these transcripts in mESC biology. Here we focused our studies on a nuclear-restricted non-coding RNA (ncRNA) that is transcribed in sense orientation from the processed, X-linked *Oct4P4*. Dramatic upregulation of sense *Oct4P4* transcription during mESC differentiation gives rise to a long, non-coding RNA that forms a complex with the repressive H3K9-specific HMTase SUV39H1. This complex translocates to the promoter of the ancestral *Oct4* protein-coding gene, located on chromosome 17, to impose H3K9 tri-methylation leading to a consecutive recruitment of HP1α and silencing of the ancestral *Oct4* gene (Fig. 6). This is in a remarkable contrast to the ceRNA function of the human cytoplasmatic lncRNA OCT4-pg4 in hepatocellular carcinoma^45^. Thus murine Oct4P4 and human *OCT4-pg4* are not mechanistically conserved and act via completely different cellular pathways. A recent study reported that anti-sense transcripts originating from the human *OCT4-pg5* can mediate silencing of the human *OCT4* gene in cancer cells^46^. Importantly, we were not able to detect anti-sense transcripts originating from murine ancestral *Oct4* or *Oct4P4* genes (Supplementary Fig. 2a). We also did not find a significant homology between the *Oct4P4* lncRNA and the *Oct4* promoter (Supplementary Fig. 7c). Finally, O*ct4P4* lncRNA did not impact on *Oct4P4* promoter activity (Supplementary Fig. 7a,b). Thus, these findings do not support a model that involves the expression of an anti-sense *Oct4P4* transcript or direct interaction of *Oct4P4* lncRNA with the *Oct4* promoter that is based on sequence homology. We rather propose that sense-oriented *Oct4P4* lncRNAs represents the functionally relevant pseudogene-derived lncRNA that forms a complex with SUV39H1 and then translocates to the promoter of the ancestral *Oct4* gene. In line with this, deleting *Oct4P4* 5′ or 3′ sequences that show high homology to the 5′UTR or 3′UTR regions, respectively, of ancestral *Oct4* significantly reduce the ability of *Oct4P4* lncRNA to deposit SUV39H1 at the Oct4 promoter, thus leading to reduced *Oct4* silencing. This highlights a possible importance of 5′UTR and 3′UTR homology regions of pseudogene-derived sense transcripts for epigenetic gene regulation.

Remarkably, the human *OCT4-pg3* (here *OCT4P3*, NR_036440.1), *OCT4-pg4* (here *OCT4P4*, NR_034180.1) and *OCT4-pg1* (here *OCT4P1b* NM_001159542.1) show homology to the entire OCT4 cDNA and share high structural and sequence homology to murine *Oct4P4*. Similar to *Oct4P4* in mESCs, we found that human *OCT4P3* lncRNA is enriched in the nuclei of OVCAR-3 and SKOV-3 ovarian cancer cells (Supplementary Fig. 8). This provides a first indication for a possible conservation of *Oct4P4/OCT4P3* function.

Interfering with the murine *Oct4P4*–SUV39H1 silencing complex in differentiated cells leads to the re-activation of mESC self-renewal transcription factor expression and telomerase-dependent telomere maintenance mechanism (Fig. 6). This represents a partial re-acqusition of mESC stem cell features in short term (6 days) *Oct4P4* lncRNA knock-down experiments. It will be interesting to test whether loss of *Oct4P4* function can increase the efficiency of iPS cell generation protocols. Together, our data indicates that *Oct4P4* and SUV39H1 are important to prevent the aberrant activation of mESCs self-renewal pathways in differentiated cells. Future experiments will aim to better define structural *Oct4P4* lncRNA motifs involved in SUV39H1 recruitment and elucidate pathways that direct *Oct4P4* to the ancestral *Oct4* gene.

Several lncRNAs such as *XIST* or *HOTAIR* have been described to act as scaffold for the formation of chromatin-modifying complexes^51^,^52^. To our knowledge, the *Oct4P4* lncRNA represents the first example for a crucial role of a vertebrate pseudogene-derived sense lncRNAs in directing chromatin-modifying activities to the promoter of its ancestral gene in *trans*.

Given the vast repertoire of vertebrate pseudogenes, we anticipate the existence of a series of pseudogene-derived lncRNAs that use mechanisms analogous to *Oct4P4* to control the expression of ancestral genes on the epigenetic level. Understanding pseudogene lncRNA-dependent mechanism of epigenetic gene regulation and a precise categorization of pseudogene-derived lncRNAs based on subcellular localization, gene expression regulation and deposition at gene promoters will provide important insights into the global role of pseudogene-derived lncRNAs in regulating higher order chromatin structures.

## Methods

### Cell culture and generation of stable cell lines

Feeder-independent mouse ESCs^59^ were cultured on gelatin (0.2%) coated plates in mESC self-renewal medium (DMEM with 15% knockout serum replacement (Gibco), 1% nonessential amino acids (Gibco), 1 mM sodium pyruvate (Invitrogen), 1% L-glutamine (Invitrogen), 0.1 mM ß-mercaptoethanol, 1% penicillin/streptomycin (Invitrogen) and 1,000 U ml^−1^ mouse leukaemia inhibitory factor. pMEFs were generated from 13.5 d.p.c C57BL/6 mouse embryos. NIH-3T3 cells were obtained from ATCC. pMEFs and NIH-3T3 cells were maintained in culture in DMEM supplemented with 10% foetal bovine serum (Lonza) and 1% penicillin/streptomycin. iPS were generated and cultivated according to standard conditions^60^. OVCAR-3 cells (ATCC) were cultured in RPMI-1640 medium supplemented with 20% foetal bovine serum (Lonza), L-glutamine (2 mM), insulin (10 μg ml^−1^; I9278, Sigma) and 1% penicillin/streptomycin. SKOV-3 cells (ATCC) and U2OS cells (ATCC) were maintained in DMEM supplemented with 10% foetal bovine serum and 1% penicillin/streptomycin. To generate stable mESC lines, cells were transfected (TransIT-LT1; Mirus Bio LCC) with respective vectors and subjected to selection using G418 (Sigma, 300 μg μl^−1^) or puromycin (Sigma, 3 μg ml^−1^).

### *Oct4* pseudogenes analysis and sequencing

Genome blast analysis ( http://blast.ncbi.nlm.nih.gov/) was performed to identify mouse genomic regions with high similarity to *Oct4* (*mus musculus Pou5f1*, transcript variant 1; NM_013633.3) mRNA. Genomic coordinates of identified pseudogenes were obtained using the University of California Santa Cruz (UCSC) genome browser. All candidate pseudogenes were PCR amplified from mESC cDNA using specific primers (Supplementary table 1). PCR amplicons were cloned into pCR2.1-TOPO vector (Life Technologies) and subjected to bi-directional sequencing.

### Oct4P4-MS2 tethering model system

To generate the *Oct4P4-24 × MS2* stem loop construct, 24 repeats of the MS2 stem loop RNA motif (obtained from pSL-MS2-24 × , ref. 61) were cloned downstream of the *Oct4P4* cDNA into pLPC. *Oct4P4* cDNA was PCR amplified using the following oligonucleotides: forward: 5′-GGGAATTCAAGCTTGTCCCTAGGTGACCAACTCCT3′-; reverse: 5′-GGGAATTCAGATCTTGTGTCCCAGGCTTTTTAAA3′-). pCMV-FLAG-MS2nls was provided by A. Marcello (ICGEB, Trieste, Italy).

### RNA immunoprecipitation

Cells were cross-linked in culture medium with 1% formaldehyde for 10 min. After addition of 125 mM glycine (PBS), cells were washed twice in ice cold PBS. Cross-linked cells were scraped in RIPA buffer (50 mM Tris-Cl, pH 7.5, 1% Nonidet P-40 (NP-40), 0.5% sodium deoxycholate, 0.05% SDS, 1 mM EDTA,150 mM NaCl) and supplemented with protease inhibitors (Complete, Roche) and RNaseOUT (Invitrogen). After incubation at 4 °C for 20 min, the cell lysate was sonicated and centrifuged. The supernatant was pre-cleared for 1 h at 4 °C with protein A/G PLUS-Agarose (sc-2003; Santa Cruz Biotechnology), supplemented with yeast transfer RNA (0.1 mg ml^−1^, Invitrogen) and incubated overnight at 4 °C with the following antibodies: mouse monoclonal anti-FLAG M2, clone M2 (2.5 μg ml^−1^; F1804; Sigma); mouse monoclonal anti-KMT1A/SUV39H1 (2.5 μg ml^−1^; ab12405; Abcam); mouse monoclonal anti-HA, (2.5 μg ml^−1^; clone HA-7, H9658; Sigma). RNA–protein complexes were recovered with protein A/G PLUS-Agarose beads and were washed six times in high-stringency RIPA buffer (50 mM Tris-Cl, pH 7.5, 1% sodium deoxycholate, 0.1% SDS, 1 mM EDTA, 1 M NaCl, 1 M urea and 0.2 mM phenylmethylsulfonyl fluoride). An aliquot of beads containing immunoprecipitatated samples were saved for western blot analysis. Remaining beads were resuspended in 50 mM Tris-Cl, pH 7.0, 5 mM EDTA, 10 mM DTT and 1% SDS and incubated at 70 °C for 45 min to reverse cross-linking. The immunoprecipitated RNA and the total RNA were extracted with the Qiazol lysis reagent (Qiagen), subjected to DNase treatment (Qiagen) and subjected to reverse transcription (Quantitect reverse transcription kit; Qiagen). The obtained cDNA used for quantitative real-time PCR (SYBR Green Universal PCR Master Mix, Applied Biosystems) on a StepOnePlus real-time PCR machine (Applied Biosystems). The oligos used for quantitative RT–PCR are reported below:

Oct4P4: forward: 5′-TGGCACCTGGCTTTAGACTTT-3′; reverse: 5′-CCAGGCCAACTTAGGGCATT-3′. 24 × MS2-Oct4P4: forward: 5′-CACCACGGCTTTGGAGTTAAG-3′; reverse: 5′-CATTAGATCTTGTGTCCCAG-3′.

Analysis of immunocomplexes by western blotting: input and immunocomplexes proteins were resuspended in SDS–PAGE sample buffer, boiled for 15 min and analysed by western blotting. Primary antibodies: mouse monoclonal anti-FLAG (1:1,000; clone M2, F1804); mouse monoclonal anti-KMT1A/SUV39H1 (1:500; ab12405); mouse monoclonal anti-HA, clone (1:1,000; HA-7, H9658) rabbit polyclonal anti-Oct4 (1:1,000; ab19857; Abcam); anti-actin (1:1,000; A2066; Sigma). Secondary antibodies coupled to horseradish peroxidase (1:1,000; A-6154; A-4416; Sigma) were used for signal detection.

### Chromatin immunoprecipitation

Cells were cross-linked in culture medium with 1% formaldehyde for 10 min, neutralized using 125 mM glycine in PBS and washed in PBS. Nuclei were obtained by lysing scraped cells in hypotonic buffer (5 mM Pipes pH 6.8, 85 mM KCl, 0.5% NP-40 and protease inhibitors), followed by centrifugation. Nuclei were resuspended in RIPA 100 mM buffer (20 mM Tris-HCl pH 7.5, 100 mM NaCl, 1 mM EDTA, 0.5% NP-40, 0.5% Na-Deoxycholate, 0.1% SDS supplemented with protease inhibitors). Chromatin was sonicated to 500—800 bp average fragment size and pre-cleared for 1 h at 4 °C with protein A/G PLUS-Agarose (sc-2003). Agarose was removed by centrifugation and an aliquot of supernatant was taken as input. Chromatin was immunoprecipitated overnight at 4 °C with the following antibodies: mouse monoclonal anti-FLAG M2, clone M2 (2.5 μg ml^−1^; F1804); mouse monoclonal anti-KMT1A/SUV39H1 (44.1; 2.5 μg ml^−1^; ab12405); rabbit polyclonal anti-trimethyl-Histone H3 (Lys9) (2.5 μg ml^−1^; 07-442; Millipore); mouse monoclonal anti-HP1a, clone 15.19s2 (2.5 μg ml^−1^; 05-689; Millipore); rabbit polyclonal anti-trimethyl-Histone H4 (Lys20) (2.5 μg ml^−1^; 07-749; Millipore); rabbit polyclonal anti-trimethyl-Histone H3 (Lys27) (2.5 μg ml^−1^; 07-449; Millipore). As a negative control for immunoprecipitation, mouse monoclonal anti-HA, clone HA-7 (2.5 μg ml^−1^; H9658) was used. DNA–protein complexes were recovered with protein A/G PLUS-Agarose beads and washed with RIPA 100 mM buffer, RIPA 250 mM buffer (20 mM Tris-HCl pH 7.5, 250 mM NaCl, 1 mM EDTA, 0.5% NP-40, 0.5% Na-Deoxycholate and 0.1% SDS), LiCl solution (10 mM Tris-HCl pH 8.0, 1 mM EDTA, 250 mM LiCl, 0.5% NP-40 and 0.5% Na-Deoxycholate) and 1 × TE. RNase treatment was performed in 1 × Tris-EDTA (TE) for 30 min at 37 °C. Cross-linking was reversed by overnight incubation at 68 °C after adding an equal volume of proteinase K solution (200 mM NaCl, 1% SDS and 0.3 mg ml^−1^ proteinase K). Samples were resuspended in ddH_2_O after phenol/chloroform extraction and ethanol precipitation. Co-immunoprecipitated DNA was analysed by RT–PCR on a StepOnePlus real-time PCR machine, using SYBR Green Universal PCR Master Mix.

The oligos used for quantitative RT–PCR are reported below.

Dkk: forward: 5′-GGGAACCAGGGAAAGAGGA-3′; reverse: 5′-GGGAAATAGGCACCCGATAA-3′. Oct4 (primer A): forward: 5′-TGCACCCCCTCCTCCTAATCC-3′; reverse: 5′-CCCTAAACAAGTACTCAACCC-3′.

Oct4 (primer B): forward: 5′-GTTGGGGGGTGGTTAGTGTCT-3′; reverse: 5′-CCACTCCTCAGTTCTTGCTTA-3′. ChIP data of Oct4 promoter regions were normalized against input and the Oct4 unrelated DKK gene.

### Transient transfection

mESCs and pMEFs were transfected with siRNAs (final concentration, 30 nM) using Lipofectamine RNAiMAX (Life Technologies) according to the manufacturer's instructions. siRNA oligonucleotide: mouse *Oct4* (Thermo Scientific Dharmacon; sense sequence: 5′-CGGAAGAGAAAGCGAACUAUU-3′), mouse *Suv39h1* (Thermo Scientific Dharmacon; sense sequence: 5′-CCAAUUACCUGGUGCAGAA-3′) mouse Oct4P4 (Thermo Scientific Dharmacon; sense sequence: 5′-GAGCAUGAGUGGAGAGGAA-3′) and mouse *Oct4P4#2* (Eurofins Genomics; sense sequence: 5′-GCCUCUCUUAAGCACUGUA-3′). siGENOME Non-Targeting siRNA#1 (D-001210-01-20, Thermo Scientific Dharmacon) and AllStars Negative Control siRNA (1027281; Qiagen; siControl#2) were used as a negative controls. Transient transfections of plasmids for pMEFs and U2OS were performed using Lipofectamine 2000 (Life Technologies) according to the manufacturer's instructions.

### Luciferase reporter assay

The mouse *Oct4* promoter, corresponding to 2.9 kb of genomic region upstream of the Oct4 ATG, was PCR amplified from mESC genomic DNA; forward 5′-GGGAATTCCTCGAGATTGTACGTAAGTACTTCAGA-3′; reverse, 5′-GGGAATTCAGATCTGGGGAAGGTGGGCACCCCGA-3′ oligonucleotides. For the mouse *Oct4P4* promoter reporter construct, 1.8 kb fragment upstream the *Oct4P4* gene was PCR amplified from mESC genomic DNA; forward: 5′-GGGAATTCCTCGAGCATATGTGTGTCAATCTTGTT-3′; reverse: 5′-GGGAATTCAGATCTGGGGAAGTTGGGCACCCCAAG-3′. Fragments were cloned into pGL3Basic (Promega) via XhoI and BglII restriction sites located upstream of the firefly luciferase gene and sequence verified. pcDNA3-HA-OCT4 is reported in ref. 22. For luciferase reporter assays, 4 × 10^4^ NIH-3T3 cells were transiently co-transfected with 400 ng of pGL3 promoter luciferase reporters and 40 ng of CMV-Renilla to normalize for transfection efficiency; when indicated, 400 ng of pCDNA3-HA-Oct4 or pCDNA3-HA vector, and 400 ng of pCDNA3-Oct4P4 or pCDNA3 vector were co-transfected. Transient transfections were performed using Lipofectamine 2000 according to manufacturer's instructions. For luciferase reporter assays involving siRNAs, plasmid DNA was transfected first followed by siRNA transfection after a 16-h recovery period. Firefly/renilla luciferase activity was assayed 72 h after transfection using the Dual Luciferase Assay kit (Promega).

### Statistical analysis

Each finding was confirmed by three independent biological replicates, unless specified. Error bars represent s.d. All *P* values were determined using two-tailed *t*-tests and statistical significance was set at *P*<0.05. The variance was similar between groups that we compared.

Uncropped scans of the most important blots are shown in Supplementary Fig. 9 in the Supplementary Information section.

## 

## Additional information

**How to cite this article**: Scarola, M. *et al.* Epigenetic silencing of Oct4 by a complex containing SUV39H1 and Oct4 pseudogene lncRNA *Nat. Commun.* 6:7631 doi: 10.1038/ncomms8631 (2015).

## Supplementary Material

Supplementary Information Supplementary Figures 1-9, Supplementary Table 1, Supplementary Methods and Supplementary Reference
