# Supplementary material for: Epigenetic silencing of Oct4 by a complex containing SUV39H1 and Oct4 pseudogene lncRNA
Source: Nat Commun. 2015 Jul 9;6:7631. doi: 10.1038/ncomms8631 (PMC4510692; doi:10.1038/ncomms8631)
Supplement: Supplementary Information — Supplementary Figures 1-9, Supplementary Table 1, Supplementary Methods and Supplementary Reference [file ncomms8631-s1.pdf]

Supplementary Figure 1

A

Name	Location	Start position	End position	Annotation	RefSeq accession number
Oct4P1	Chr 3	129433673	129435000	Mus musculus predicted gene 5712 (Gm5712), non-coding RNA	NR_033594.1
Oct4P2	Chr 1	159293805	159294189	N.A.	-
Oct4P3	Chr 14	17105602	17106308	N.A.	-
Oct4P4	Chr X	52878731	52880395	Mus musculus predicted gene 6539 (Gm6539), non-coding RNA	NG_021129.1
Oct4P5	Chr 6	103964077	103964639	N.A.	-

B

Oct4P1 (182 bp)
...GGCGATTGGGCCCTCTAGATGCATGCTCGAGCGGCCGCCAGTGTGATGGATATCTGCAGAATTCGCCCTTGGGAATTCGACGTCGGGATGCTGTGAGCCAAGGCAAGGAAGAGAGGTA
GACAAGAGAACCTGAAGTTTTGGGGTTAAATTCTTTTACTGAGGAGGGATTAAAAGCACAAAGGGGGTGGGGTGGGGATGGGATAGGATAAGAAGTTCAGTGATGCTGTTGATCTGGAGC
CTGTCTGTCACTCATCCTTAAATAAAGAATTCCCAAGGGCGAATTCCAGCACACTGGCGGCCGTTACTAGTGGATCCGAGCTCGGTACCAAGCTTGGCGTAATCATGGTCATAGCTGTTTCC
TGTGTGAAATTGTTATCCGC...

Oct4P2 (394 bp)
...TAGGGCGATTGGGCCCTCTAGATGCATGCTCGAGCGGCCGCCAGTGTGATGGATATCTGCAGAATTCGCCCTTGGGAATTCTTTAAGAACAAAATGATGAGTGACAGACAGGCCAGGCT
CCTGATCAACAGCATCACTGAGCTTCTTTCCCCATCCCACCCCCCACCCCTGTTGTGCTTTTAATCCCTCCTCAGTAAAAGAATTTAACCCCAAAGCTCCAGGTTCTCTTGTCTACCTCCCTT
GCCTTGGCTCACAGCATCCCCAGGGAGGGCTGGTGCCTCAGTTTGAATGCATGGGAGAGCCCAGAGCAGTGACGGGAACAGAGGGAAAGGCCTCGCCCTCAGGAAAAGGGACTGAGT
AGAGTGTGGTGAAGTGGGGGCTTCCATAGCCTGGGGTGCCAAAGTGGGGACCTGGGGGCAGAGGAAAGGATACAGCCCCCCCTGTCTTCTACACTGGGTGAAACTTGAGCATACGGAAT
TCCCAAGGGCGAATTCCAGCACACTGGCGGCCGTTACTAGTGGATCCGAGCTCGGTACCAAGCTTGGCGTAATCATGGTCATAGCTGTTTCCTGTGTGAAATTGTTATCCGCTCACAATTCC
ACACAACATA...

Oct4P3 (214 bp)
...AGGGCGATTGGGCCCTCTAGATGCATGCTCGAGCGGCCGCCAGTGTGATGGATATCTGCAGAATTCGCCCTTTGTCACTACCCTGGCTCTCCCATGCATTCAGACTGAGGCACCAGCAC
TCCCTGGGGATGATGTGAACTAAGGCAAGGAAGGGAGGTAGACAAGAGAACCCGGAGCTTTGGGGTTAAATTCTTTTATTGAGGAGGGATTAAAAGCACAACAGGGGTGGGCATGGGATA
AGAAGTCCAGTGATGCTGTTGATCAGGAGCTCGTCCATCACTCATCAAAGGGCGAATTCCAGCACACTGGCGGCCGTTACTAGTGGATCCGAGCTCGGTACCAAGCTTGGCGTAATCATG
GTCATAGCTGTTTCCTGTGT...

Oct4P4 (160 bp)
...CGATTGGGCCCTCTAGATGCATGCTCGAGCGGCCGCCAGTGTGATGGATATCTGCAGAATTCGCCCTT**C**TGGCACCTGGCTTTAGACTTTATCTTCTTACTCCCATAGGGAGTGGGGAGG
ATGGGTAAGGAAAGCTGGAGCTGGGCTGGGTGGACCCTTTGACCTGGATAAGCTCCCAAGGGCCCCCAAGTGGGCCTGGAATGGGGCCAGAGGGTGGACAAGGCTTGGAAGGGCGAA
TTCCAGCACACTGGCGGCCGTTACTAGTGGATCCGAGCTCGGTACCAAGCTTGGCGTAATCATGGTCATAGCTGTTTCCTG...

Oct4P5 (155 bp)
...AGGGCGATTGGGCCCTCTAGATGCATGCTCGAGCGGCCGCCAGTGTGATGGATATCTGCAGAATTCGCCCTTAGCTCCCATAGCCTGGGGTATCAAAGTGTCAAAGTGGGGACCTGGG
GGCAGAGGAAAGGATACAGCCCCCCCTCCCCCCCGCAAGGCGTCCCTGTGACCTCATACTCCTCTCGTTGGGAATACACAATGTTTGATCGTTGGCCCTTCTGGCACCGAAGGGCGAATT
CCAGCACACTGGCGGCCGTTACTAGTGGATCCGAGCTCGGTACCAAGCTTGGCGTAATCATGGTCATAGCTGTTTCCT...

C

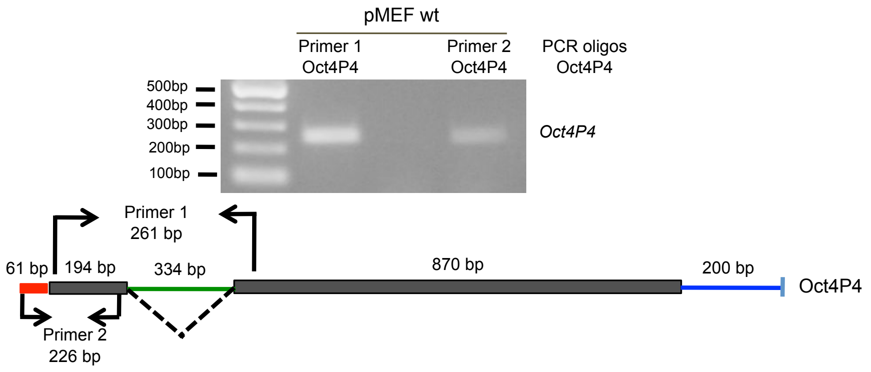

D

Oct4P1

Met A G H L A S D F A F S P H Q V G A Met G Q Q G W S R A G W T L E P G **Stop** A S K G L W V G L E Stop D Q A **Stop** R Y W D L P **Met** S T G I **Stop** V L R R D G I L W T S G W R G D
F A A **Stop** E P G K S R C G E Q L R G S L L W A L C R P P Q C R E V G D E G T T S R G V P G H E S P A E G A R E V C Q A A E A E E D H L G V H P D R R G A H P G R S L Q K G V S
Q T T I Y C F E A R Q L S L **Met** T Met H K L R P L L E K W V E E A E D N E N L Q E I C K S E T Met V Q A W K K K S A S I E N H V R Q S L E N E F L K C P E P S P Q Q I T S I S I T Met
Q L G Met E K D Met V R G W F C N R R Q K G K R S S I E Y S Q R E K Y E A F R G C L S G R G V G L Y P F L C P Q A P L W H P K L W E P P L H H T T R Y L S **Stop** G R G L S L C P
L S L L W A L P C I Q T E T P A L P G D A V S Q G K E E R **Stop** T R E P E V L G L N S F T E E G L K A R R G W G G D G I G **Stop** E V Q **Stop** C C **Stop** S G A C L S L I L K **Stop** R L G
H T V D S

Oct4P2

Q G G L Y P F L C P Q V P T L A P Q A **Met** E A P T S P H S T Q S L F L R A R P F P L F P S L L W A L P C I Q T E A P A L P G D A V S Q G K G G R Q E N L E L W G **Stop** I L L L R R D
Stop K H N R G G G W D G E R S S V **Met** L L I R S L A C L S L I I L F L N K D W D T Q **Stop**

Oct4P3

G V Q P D N H L S L **Stop** G P A A C V S C G P F **Stop** R S W W R K P P T R R E P L G N **Met** Q I G G P G A G W E E N S S S I E N N V R W S L E N V F L K C P K S S L Q Q I T S I A K
Q L G L V K D V V R V W F C N W H Q N G K R S S T E Y S Q P E E F E I Q G H L Y W G V G V G V G A V S F P L P Q V P T L A P Q A **Met** G D P T S P H S T Q S L F L W A R P F P L F
L S L P W L S H A F R L R H Q H S L G **Met Met Stop** T K A R K G G R Q E N P E L W G **Stop** I L L L R R D **Stop** K H N R G G H G I R S P V **Met** L L I R S S S I T H H F Y S **Stop** I K T

Oct4P4

Met A W H L A L D F I F L L P **Stop** G V G R **Met** G K E S W S W A G W T L **Stop** P G **Stop** A P K G P Q V G L E W G Q R V D K A W G C W G S P H I P Q S S **Met** E E E **Stop** H I V N L
R L D W D K C P K L A W R L C S L R P S W S **Met** S G E E L R E G L S **Stop** A L Y S P P E C C E V G E A G T K S P G V S G H E S P A D R T R V V C Q T A E A E E D Q F G V Q P G Q
H G A H P G C S L W R S V Q P D H H L Q L **Stop** G P A A Q L Q E L V **Stop** T A A P A G E V G G G S G Q Q **Stop** G C S R **Met** Y C K P W C R P A R E S R **Stop** A L R T L R R G T **Stop**
K T H F C S G G S L P C S R S A A S S N S L G W R R **Met** W P G C G S V T C T R R V N K **Stop** S S I D Y S Q Q V R S C R A S F G G D L Y P F L C F Q G L I L V P R G **Met** G T P I S P
H C T H W S L F A R I R P L P L F L S L L **Stop** A L P C I Q A E A T S L P G G E V S L G E E G G **Stop** R E P G A T T A L E L S S L T E E R F K A Q K G **Stop** R G R **Stop** S Y A V E C E L
S L T I L F L N L K S L G H

Oct4P5

T **Stop** K R K W T S T E N C L R **Stop** S L E N **Met** F Q K C R K P S L Q E I T S I I T K Q L R L E K D V A R V W F C T R C Q K G Q R S N I V Y S Q R E E Y E V T G T P C G G E G G L Y
P F L C P Q V P T L T L **Stop** Y P R L W E L P L H H T L L S P F S **Stop** G **Stop** G L P F V P P H C Y G A L P C I Q A **Stop** A P A L P G S D V S L G K G G R **Stop** T K R T W S S G V T F F
Y **Stop** T R I K S T R L G G R G G I R S S V **Met**

E

	Oct4P4 (molecules/μg of RNA)	Absolute Fold (ES set as 1)	Relative Fold (pMEF RNA/ cells= 4.8x ES RNA per cell) ES set 1
ES cells	6882.29	1	1
pMEF cells	906741.14	131.74	632

	Oct4 (molecules/μg of RNA)	Absolute fold (ES set as 1)	Relative fold (pMEF RNA/ cells=4x ES RNA per cell)
ES cells	51617142.8	-	-
pMEF cells	N.D.	-	-

(A) Chromosomal localization, genomic coordinates (*UCSC* genome browser), annotation and *RefSeq* accession numbers of *Oct4* pseudogenes describe in this study. Only *Oct4P1* and *Oct4P4* result currently annotated. **(B)** Sequencing validation of *Oct4* pseudogenes transcripts addressed in this study. Individual *Oct4* pseudogene cDNA templates where specifically amplified by RT-PCR and subcloned before sequencing. **(C)** Analysis of *Oct4P4* transcripts in mouse embryonic fibroblast (MEF) by semi-quantitative RT-PCR using specific primers identifying spliced and non-spliced *Oct4P4* mRNA. Top panel: *Oct4P4* RT-PCR products visualized on an 1.2% agarose gel. Bottom panel: schematic representation of possible PCR products. Primer pair 1 gives a 261bp product indicative for Oct4P4 pr-mRNA splicing. Primer pair 2 amplifies a 226bp region upstream the spliced insert, demonstrating that *Oct4P4* transcription is initiated upstream of the spliced insert. **(D)** Potential peptide sequences encoded by Oct4 pseudogenes. Potential start codons (Met) are indicated. Stop codons (Stop) are indicated. The maximum ORF predicted for *Oct4P4* spans 27 amino acids. **(E)** Estimated number of *Oct4* and *Oct4P4* molecules per microgram of total RNA in mESC and pMEFs. *Oct4* was not detectable in pMEF. Absolute fold: difference of total Oct4P4 (upper panel) or Oct4 (bottom panel) mRNA levels per μg of total RNA in pMEFs when compared to mESCs (set as 1). Relative fold: pMEFs (4.8 pg RNA/cell) contain 4.8 fold more tatal RNA than mESCs (1pg RNA/cell). Indicated values correct for differences in total RNA in mESCs compared to pMEFs. pMEFs cotain 632 fold more *Oct4P4* than mESCs.

Supplementary Figure 2.

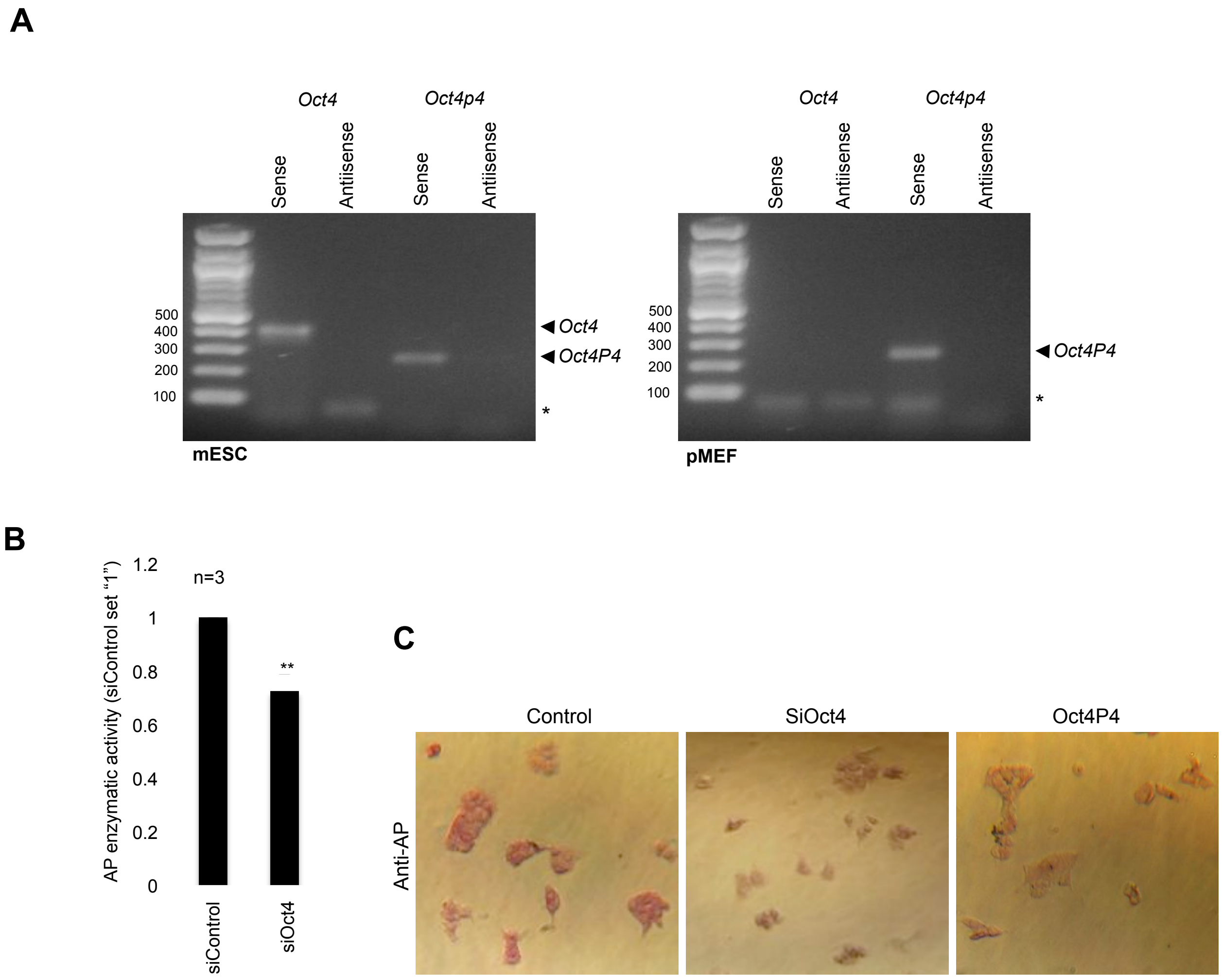

(A) Strand specific *Oct4* and *Oct4P4* PCR products obtained from mESC and pMEF cDNA, run on a 1.2% agarose gel. Asterisk indicates primer dimers. *Oct4* was not detectable in pMEF.**(B)** Alkaline phosphatase (AP) activity in mESCs after RNAi mediated depletion of *Oct4.* **(C)** AP staining of mESC colonies transfected with the indicated siRNA or expression vector. siOct4 and *Oct4P4* overexpression reduce AP activity.

Supplementary Figure 3.

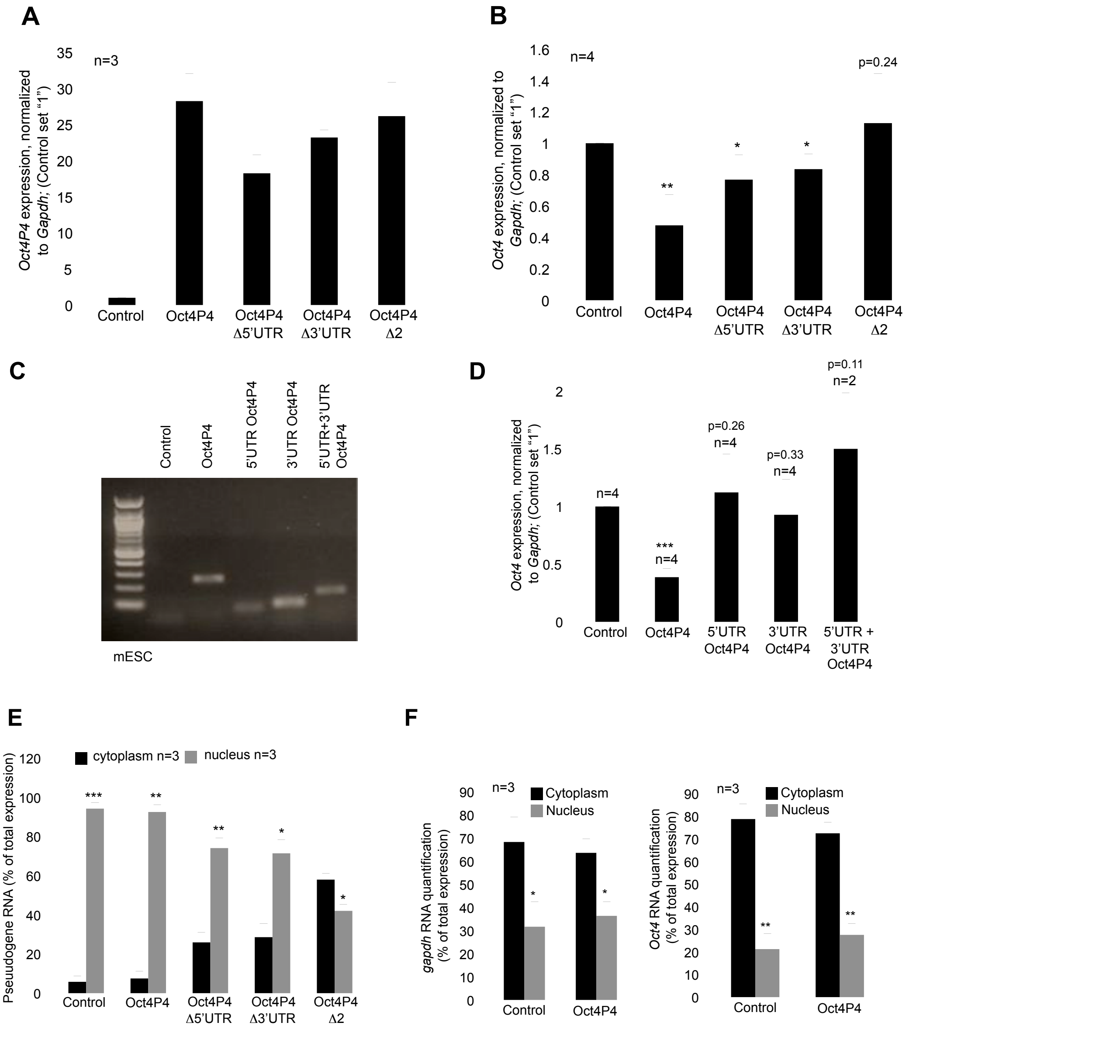

(A) Quantitative RT-PCR analysis using mESCs stably expressing Oct4P4, Oct4P4Δ5'UTR, Oct4P4Δ3'UTR or Oct4P4Δ2. Expression values were normalized against *Gapdh*. **(B)** Quantitative RT-PCR analysis of ancestral *Oct4* using mESCs stably expressing Oct4P4, Oct4P4Δ5'UTR, Oct4P4Δ3'UTR and Oct4P4Δ2. *Oct4* expression values were normalized against *gapdh*. Ectopic Oct4P4Δ5'UTR or Oct4P4Δ3'UTR reduce ancestral *Oct4* expression less efficiently than full length *Oct4P4*. Ectopic Oct4P4Δ2 does not impact on *Oct4* expression. **(C)** Oct4P4, Oct4P4-5'UTR, Oct4P4-3'UTR orOct4P4-5'UTR+3'UTR (fusion of Oct4P4-5' and 3'UTRs) specific RT-PCR products were run on a 1.2% agarose gel. **(D)** Quantitative RT-PCR analysis using mESCs stably expressing Oct4P4, Oct4P4-5'UTR, Oct4P4-3'UTR or Oct4P4-5'UTR+3'UTR (fusion of Oct4P4-5' and 3'UTRs). *Oct4* expression values were normalized against *gapdh*. Expressing exclusively the 5' and or the 3' UTR of *Oct4P4*, does not result in repression of *Oct4*. **(E)** Quantitative real-time PCR demonstrating subcellular localization of Oct4P4Δ5'UTR, Oct4P4Δ3'UTR

orOct4P4Δ2 in mESCs. Expression values are shown as percentage of total expression. Ectopic Oct4P4Δ5'UTR or Oct4P4Δ3'UTR show reduced nuclear localization when compared to *Oct4P4*. Ectopic Oct4P4Δ2 shows almost equal distribution between nucleus and cytoplasm. **(F)** Quantitative real-time PCR indicating subcellular localization of *gapdh* and *Oct4* in mESCs stably overexpressing *Oct4P4* or a control construct. Expression values are shown as percentage of total expression. Ectopic *Oct4P4* expression does not cause changes of *gapdh* or *Oct*4 mRNA localization. . n, refers to the number of independent experiments carried out; error bars indicate standard deviation; a Student's t-test was used to calculate statistical significance values: *, $p < .05$; **, $p < .01$; ***, $p < .001$.

Supplementary Figure 4.

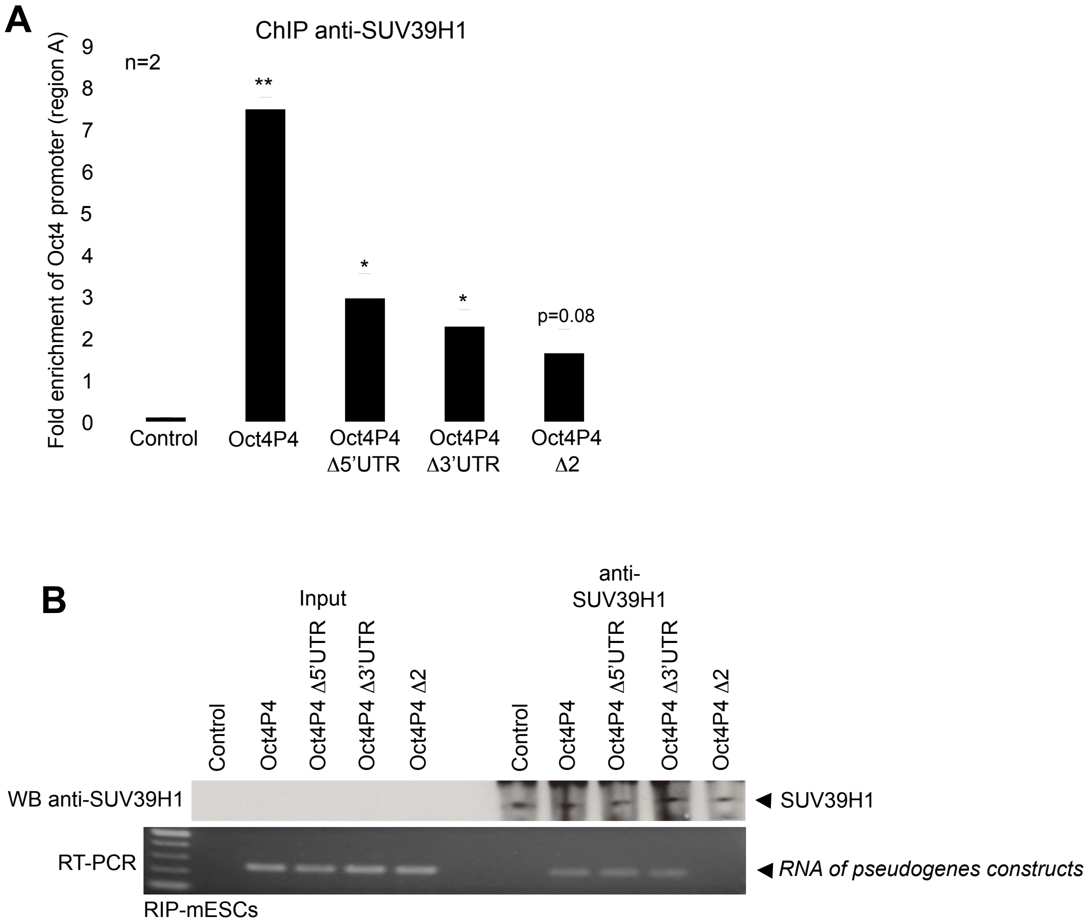

(A) *Oct4* promoter ChIP analysis using SUV39H1 specific antibodies using mESCs transiently transfected with the indicated constructs. Upon deletion of the 5' or the 3' UTR of *Oct4P4*, binding of SUV39H1 to the *Oct4* promoter is reduced. Oct4P4Δ2 does not interact with SUV39H1. **(B)** Anti-SUV39H1 RIP using mESCs overexpressing Oct4P4, Oct4P4Δ5'UTR, Oct4P4Δ3'UTR or Oct4P4Δ2 mESCs. Wild type mESCs were used as control. Immunoprecipitation of SUV39H1 was validated by western blotting (top). Agarose gel electrophoresis after semiquantitative PCR demonstrates the presence of RNA of pseudogenes in anti-Suv39h1 RIP experiments (bottom). Deletion of *Oct4P4* 5'UTR and 3'UTR homology renders *Oct4P4* unable to interact with SUV39H1. Endogenous *Oct4P4* (control) expression levels are to low in mESC to permit *Oct4P4* detection in SUV39H1 immunoprecipitations. n, refers to the number of independent experiments carried out; error bars indicate standard deviation; a Student's t-test was used to calculate statistical significance values: *, $p < .05$; **, $p < .01$.

Supplementary Figure 5.

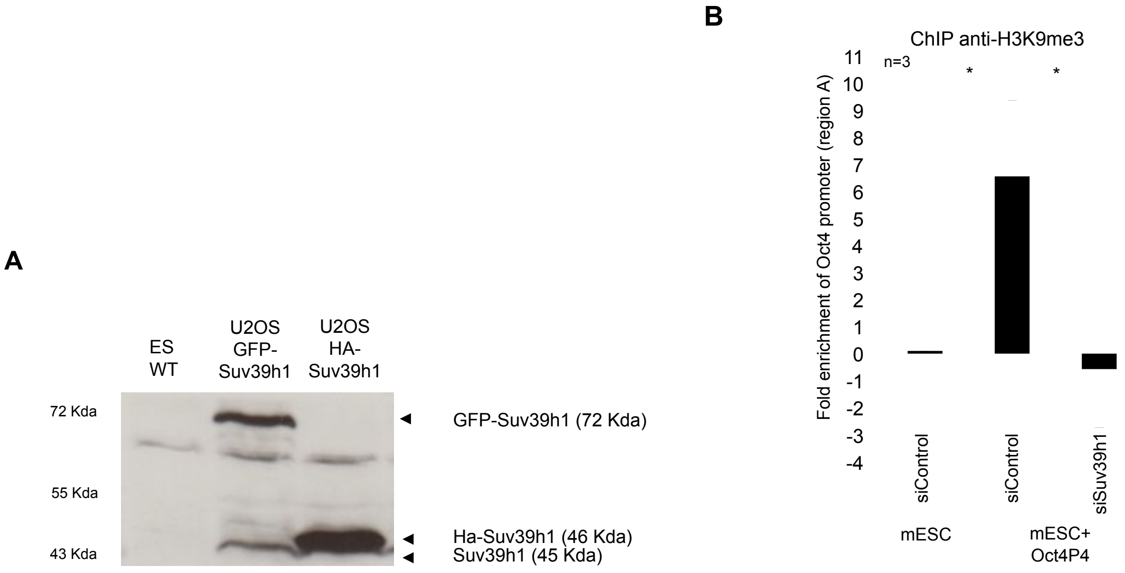

(A) SUV39H1 antibody specificity in western blotting experiments. Endogenous SUV39H1 (45 kDA) is undetectable in mESCs, probably due to low expression levels. SUV39H1 antibody detects specific bands in western blotting experiments using U2OS cells transiently transfected with GFP-tagged Suv39h1 (72 kDa) or HA-tagged SUV39H1 (46 kDa). This confirms that the SUV39H1 antibody used in this study specifically detects SUV39H1 in the context of SUV39H1 enrichment such as SUV39H1 overexpression or immunoprecipitation (see Fig. 4a, b). **(B)** *Oct4* promoter ChIP analysis using H3K9me3 specific antibodies was performed in *Oct4P4* overexpressing mESCs. Oct4P4 overexpressing mESCs were transiently transfected with indicated siRNAs. Knock-down of SUV39H1 decreases abundance of H3K9me3 at the promoter of the ancestral *Oct4* gene. n, refers to the number of independent experiments carried out; error bars indicate standard deviation; a Student's t-test was used to calculate statistical significance values: *, $p < .05$.

Supplementary Figure 6.

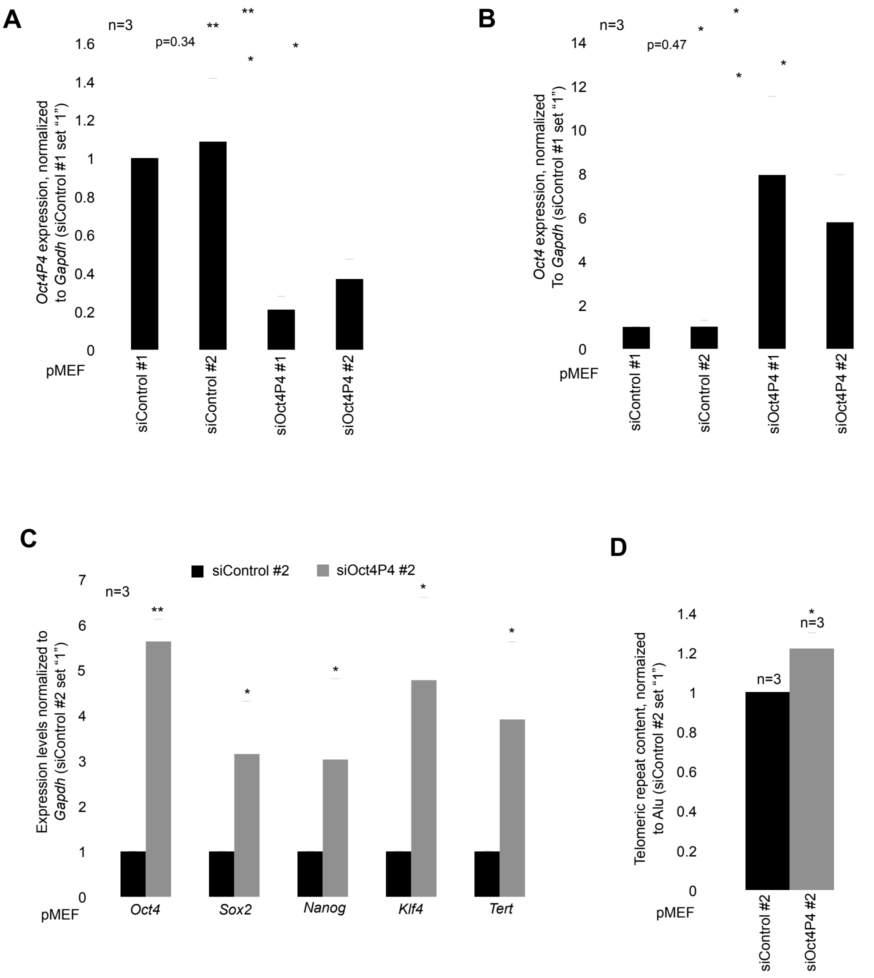

(A) Quantitative RT-PCR determining *Oct4P4* expression levels in pMEFs transiently transfected with two different siRNAs (#1, #2) targeting *Oct4P4.* Control cells were transfected with two different siRNA control oligos. *Oct4P4* expression was normalized to *gapdh.* Both *Oct4P4* specific RNAi oligonucleotides (#1, #2) significantly decrease *Oct4P4* expression levels. **(B)** Quantitative RT-PCR determining *Oct4* expression levels in pMEFs transiently transfected with two different siRNAs (#1, #2) targeting *Oct4P4.* Control cells were transfected with two different siRNA control oligos (#1, #2). *Oct4P4* expression was normalized to *gapdh.* Both *Oct4P4* specific siRNAs significantly increase ancestral *Oct4* expression. **(C)** Quantitative RT-PCR determining mESCs self-renewal marker genes expression levels in pMEFs transiently transfected with indicated siRNAs. siOct4P4#2 signficantly increases ancestral Sox2, Nanog, Klf4 and hTERT expression. These results reproduce data from Fig. 2G **(D)** Measurement of telomere repeat content by quantitative RT-PCR. Total genomic DNA from pMEFs transiently transfected with indicated RNAi oligonucleotides subjected to quantitative RT-PCR using telomere specific PCR primers. Alu-equivalent B1 repeats were used as reference sequence. Transfection of siOct4P4#2 increases telomere repeat content. n, refers to the number of independent experiments carried out; error bars indicate standard deviation; a Student's t-test was used to calculate statistical significance values: *, $p < .05$; **, $p < .01$

Supplementary Figure 7.

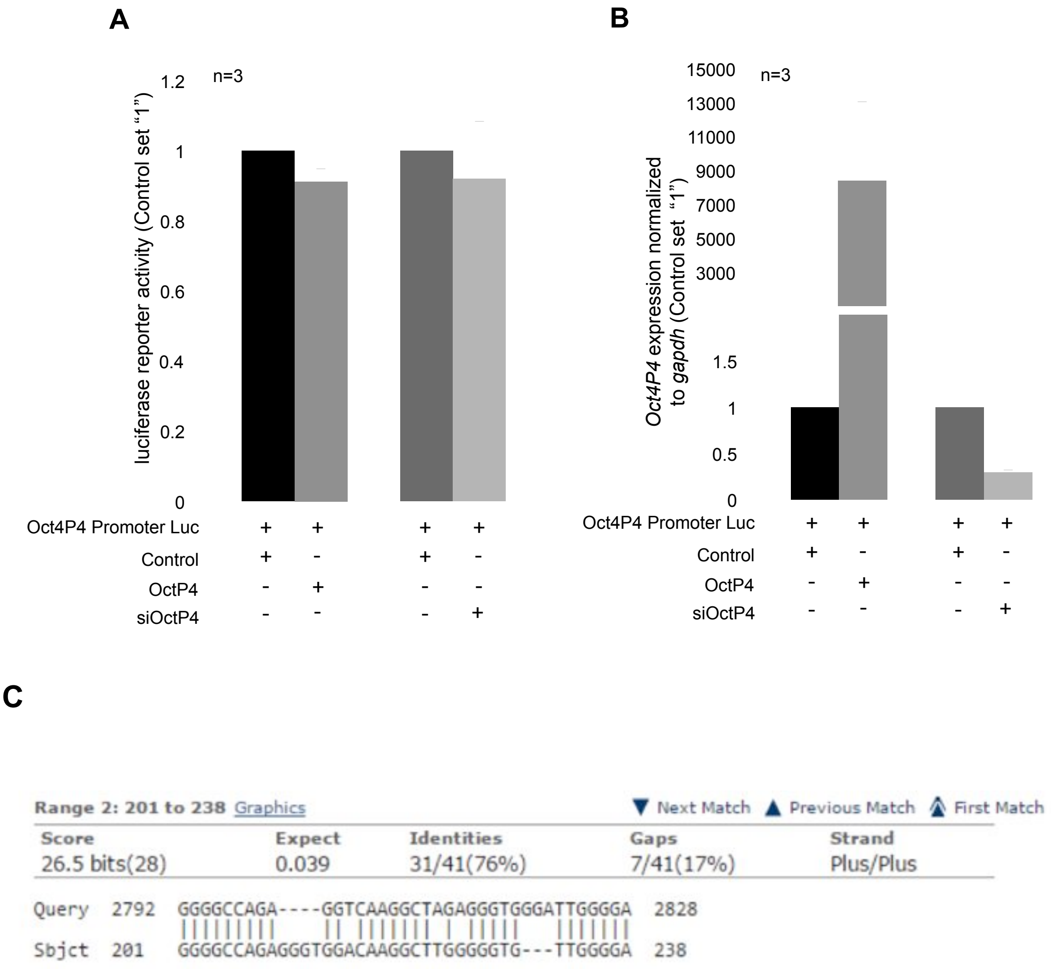

(A) *Oc4P4* promoter luciferase reporter assay. NIH-3T3 cells were co-transfected with indicated vectors and siRNAs. Luciferase activity was assayed 72h after co-transfection. *Oct4P4* does not impact on Oct4P4 promoter activity. **(B)** Quantitative RT-PCR validating *Oct4P4* expression levels in NIH-3T3 cells previously co-transfected with indicated vectors or siRNAs. **(C) Blast alignment between 5' UTR of *Oct4P4* and the Oct4 promoter.** n, refers to the number of independent experiments carried out; error bars indicate standard deviation; Student's t-test calculations did not reveal statistical significant results (p > .05).

Supplementary Figure 8.

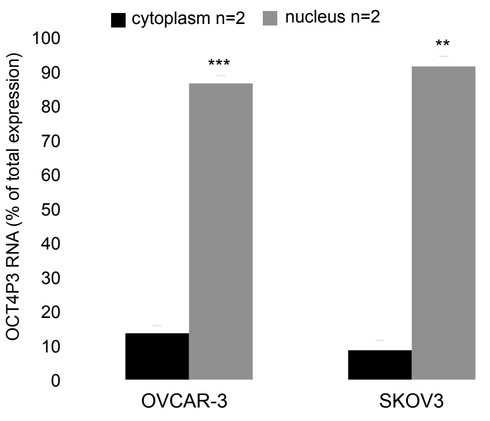

Subcellular localization of the human OCT4 pseudogene 3 (*OCT4P3*) lncRNA. Percentage of total RNA found in the nuclear and cytoplasmic fractions of ovarian cancer cell lines OVCAR-3 and SKOV-3, as determined by quantitative real time (RT-PCR). OCT4P3 lncRNA shows prevalent nuclear localization.

Supplementary Figure 9.

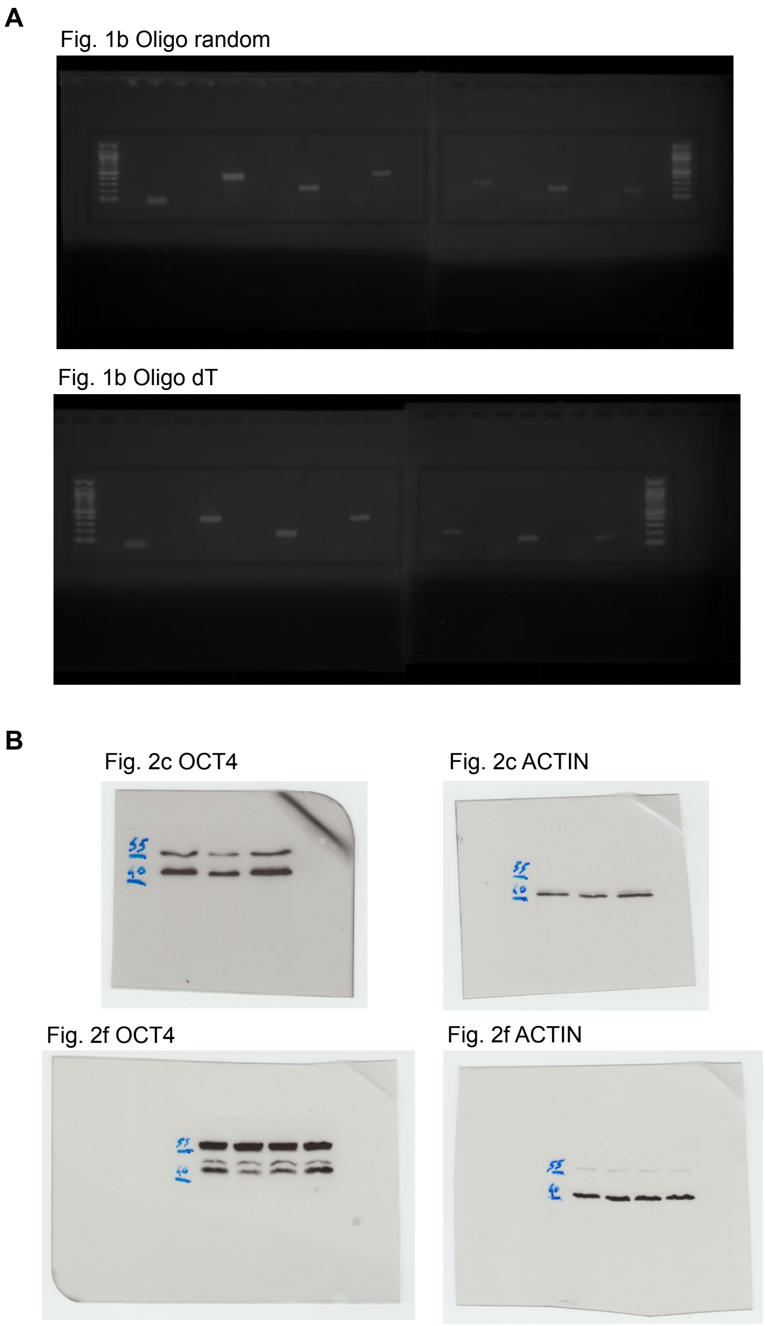

(A) Uncropped gels from Figure 1. **(B)** Uncropped blots from Figure 2. Images of the selected portions shown in Figure 1 and Figure 2 are indicated.

C

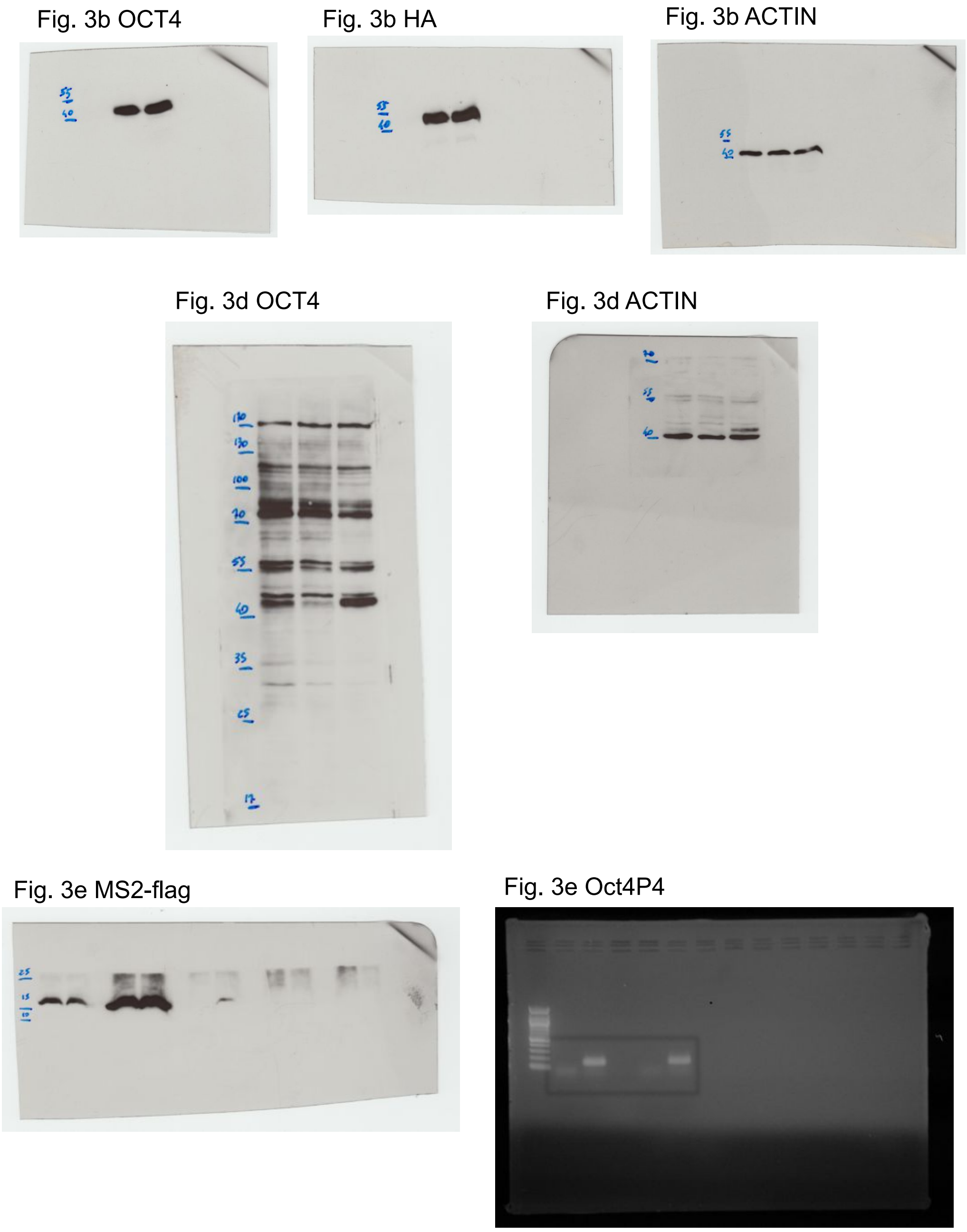

(C) Uncropped blots and gels from Figure 3. Images of the selected portions shown in Figure 3 are indicated.

D

Fig. 4a SUV39H1

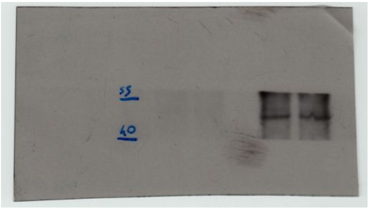

Fig. 4a MS2-flag

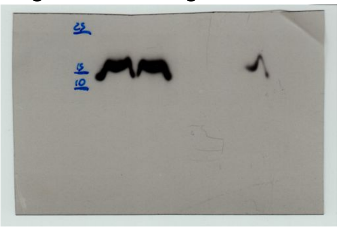

Fig. 4a Oct4P4

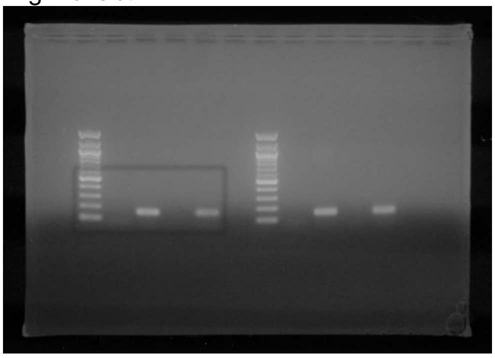

Fig. 4b SUV39H1

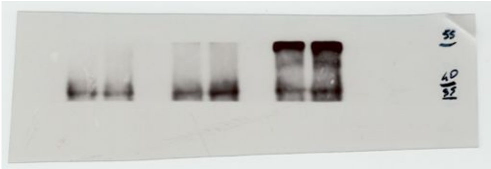

Fig. 4b Oct4P4

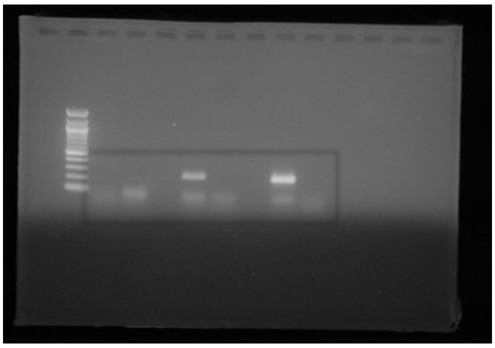

Fig. 4c OCT4

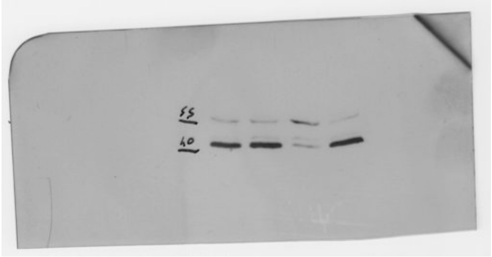

Fig. 4c ACTIN

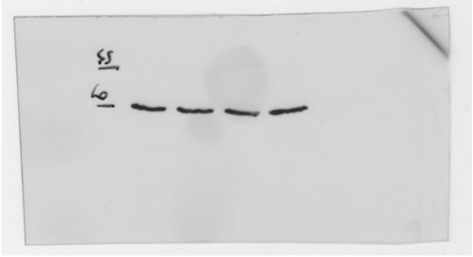

(D) Uncropped blots and gels from Figure 4. Images of the selected portions shown in Figure 4 are indicated.

Supplementary Table 1

PCR Primers

**Name**	**Sequence (5’-3’)**
Brachyury f	CAGCCCACCTACTGGCTCTA
Brachyury r	GAGCCTGGGGTGATGGTA
Fgf5 f	GTTTCCAGTGGAGCCCTT
Fgf5 r	GAGACACAGCAAATATTTCCAAAA
Gapdh f	TTCACCACCATGGAGAAGGC
Gapdh r	CCCTTTTGGCTCCAC
Gdf3 f	TCAGCTTCTCCCAGACCAGGGTTT
Gdf3 r	CACACGCCCCGGTCCTGAAC
Klf4 f	CCGGTCCCTAGAGGCCCATTT
Klf4 r	AGTTCATCGGAGCGGGCGAA
Nanog f	TTCTTGCTTACAAGGGTCTGC
Nanog r	AGAGGAAGGGCGAGGAGA
Nestin f	CTGCAGGCCACTGAAAAGTT
Nestin r	GACCCTGCTTCTCCTGCTC
Oct4 f	CAGGGACACCTTTCCCAGGG
Oct4 r	TTTAAGAACAAAATGATGAG
Oct4P1 f	GGGATGCTGTGAGCCAAG
Oct4P1 r	TTTATTTAAGGATGAGTGAC
Oct4P2 f	CGTATGCTCAAGTTTCAC
Oct4P2 r	TTTAAGAACAAAATGATGAG
Oct4P3 f	TGTCACTACCCTGGCTCTC
Oct4P3 r	TGATGAGTGATGGACGAGC
Oct4P4 f	TGGCACCTGGCTTTAGACTTT
Oct4P4 r	CCAGGCCAACTTAGGGCATT
Oct4P5 f	CGGTGCCAGAAGGGCCAACG
Oct4P5 r	AGCTCCCATAGCCTGGGGTATCA
Otx f	AGCTGCTCCCTCCGAAGCAGTA
Otx r	AGCCGGACGGTCTCGATTCG
Sox2 f	TGCTGCCTCTTTAAGACTAGGG
Sox2 r	TCGGGCTCCAAACTTCTCT
Suv39h1 f	CGGATCACCGTGGAGAAT
Suv39h1 r	CACTCACAGCCAACAGCTACCT
Tert f	GCAGGTGAACAGCCTCCAGACAG
Tert r	TCCTAACACGCTGGTCAAAGGGAAGC
Oct4P4 semi-qPCR f	CTGGCACCTGGCTTTAGACTTT
Oct4P4 semi-qPCR r	CCAAGCCTTGTCCACCCTCTG
Oct4P4 primer 1 f	CTGGCACCTGGCTTTAGACTTT
Oct4P4 primer 1 r	CCAGGCCAACTTAGGGCATT
Oct4P4 primer 2 f	GTCCCTAGGTGACCAACT
Oct4P4 primer 2 r	CCAAGCCTTGTCCACCCTCTG
OctP4-5’UTR f	GTCCCTAGGTGACCAACT
OctP4-5’UTR r	GGGGAAGTTGGGCACCCC
OctP4-3’UTR f	TAGAGAGGAAGATGAAGT
OctP4-3’UTR r	TGTGTCCCAGGCTTTTTA
OctP4-5’UTR-3’UTR f	GTCCCTAGGTGACCAACT

OctP4-5'UTR-3'UTR r	TGTGTCCCAGGCTTTTTA
hOCT4P3 f	CTTCGGATTTCGCCTTCTCA
hOCT4P3 r	GGGCACTAGCCCCACTCCAGT

Supplementary Methods:

Plasmids

pCDNA3-Oct4P4 was generated by amplifying a genomic region encoding *Oct4P4* from mESCs by PCR (Forward: GGGAATTCCTCGAGGTCCCTAGGTGACCAACTCCT; Reverse: GGGAATTCTCTAGATGTGTCCCAGGCTTTTTAAAT) and cloning into pCDNA3 vector (Promega) via XhoI and XbaI restriction sites. pcDNA3-Oct4p4Δ1 was generated by amplifying the corresponding region by PCR (Fragment 1: Forward: GGGAATTCCTCGAGGTCCCTAGGTGACCAACTCCT ; Reverse: GGGAATTCGAATTCCTGGCCCCATTCCAGGCCCA. Fragment 2: Forward: GGGAATTCGAATTCCCTGGAGACTTTGCAGCCTTA; Reverse: GGGAATTCTCTAGATGTGTCCCAGGCTTTTTAAAT) and insertion into XhoI and XbaI sites of pCDNA3 (Promega). pcDNA3-Oct4P4Δ2 was obtained by amplifying the relevant genomic region by PCR (Forward: GGGAATTCCTCGAGATGGCCTGGCACCTGGCTTTAG; Reverse: GGGAATTCTCTAGACCCTTTCTGTGCTTTGAACCT) and cloning into XhoI and XbaI sites of pcDNA3 (Promega). pcDNA3-Oct4P4Δ5'UTR and pCDNA3-Oct4P4Δ3'UTR were generated by amplifying the corresponding regions by PCR (pcDNA3-Oct4P4Δ5'UTR: Forward: GGGAATTCCTCGAGATGGCCTGGCACCTGGCTTTAG; Reverse: GGGAATTCTCTAGATGTGTCCCAGGCTTTTTAAAT) (pCDNA3-Oct4P4Δ3'UTR: Forward: GGGAATTCCTCGAGGTCCCTAGGTGACCAACTCCT; Reverse: GGGAATTCTCTAGACCCTTTCTGTGCTTTGAACCT) and cloning into pcDNA3 (Promega) via XhoI and XbaI restriction sites.

pcDNA3-Oct4P4-5'UTR was obtained by PCR amplifying the 5'UTR region of Oct4P4 (Forward: AAGCTTGTCCCTAGGTGACCAACT; Reverse: CTCGAGGGGGAAGTTGGGCACCCC) and insertion into HindIII and XhoI sites of pcDNA3 vector (Promega). pcDNA3-Oct4P4-3'UTR was obtained by amplifying the 3'UTR of Oct4P4 by PCR (Forward: CTCGAGTAGAGAGGAAGATGAAGT; Reverse: TCTAGATGTGTCCCAGGCTTTTTA) and cloning into XhoI and XbaI sites of pcDNA3 (Promega). **pcDNA3-Oct4P4-5'UTR-3'UTR** was PCR-amplified from the corresponding

region of Oct4P4 (Fragment 1: Forward: AAGCTTGTCCCTAGGTGACCAACT; Reverse: CTCGAGGGGGAAGTTGGGCACCCC. Fragment 2: Forward: CTCGAGTAGAGAGGAAGATGAAGT; Reverse: TCTAGATGTGTCCCAGGCTTTTTA) and inserted into pcDNA3 (Promega) via HindIII and XbaI .

For Suv39h1 constructs, Suv39h1 was PCR-amplified from mESC cDNA (pLPC-HA-Suv39h1: Forward: GGATCCATGGCGGAAAATTTAAAAGGT; Reverse: CTCGAGCTAGAAGAGGTATTTTCGGC) (pLPC-GFP-Suv39h1: Forward: GAATTCATGGCGGAAAATTTAAAAGGT; Reverse: CTCGAGCTAGAAGAGGTATTTTCGGC) and cloned into pLPC-HA vector via BamH1 and XhoI, and into pLPC-GFP vector via EcoRI and XhoI restriction sites.

Quantitative determination of *Oct4P4* and *Oct4* RNA molecules

To estimate the copynumber of endogenous *Oct4P4* and *Oct4* transcripts, vectors containing subcloned fragments of Oct4P4 and Oct4 (pTOPO-Oct4P4 and pGL3-3'UTR-Oct4 were used as internal standards for copy number (Supplementary Reference [1]). 0.5 µg of total RNA from mESCs and pMEFs was subjected to reverse transcription using the Quantitect reverse transcription kit (Qiagen) according to the manufacturer's protocol.

Equal volumes (5µl) of target cDNA and serially dilutions of vectors (mol/µl; external standard) were used in quantitative RT-PCR in order to determine the molar range of the two target RNAs. To more precisely define the number of *Oct4* and *Oct4P4* molecules in 5µl of cDNA, limited molar dilutions of vectors (internal standard) were then added to target cDNA in each quantitative RT-PCR reaction. This enables us to determine the number of *Oct4/Oct4P4* mRNA molecules in each reaction tube. The number of molecules obtained for *Oct4P4* and *Oct4* was expressed as molecules per µg of total RNA used for RT.

Embryoid body differentiation

For mESC differentiation medium (Dulbecco's Eagle's medium (DMEM) supplemented with 15% ES cell certified serum (Invitrogen), 1% non-essential amino acids (Gibco), 1mM sodium pyruvate (Gibco), 1% L-glutamine (Invitrogen), 0.1 mM ß-mercaptoethanol and 1% penicillin/streptomycin (Invitrogen) was used. 300 mESCs were cultured in hanging drops for three days. After 3 days EBs were transferred to low-attachment 24-well plates (Euroclone) and grown in suspension for 4 days.

Alkaline phosphatase assays

mESC colonies were stained using the StemTAG Alkaline Phosphatase Staining Kit (CBA-302; Cell Biolabs) following the manufacturer's instructions. Self-renewal colonies stain positive for alkaline phosphatase (AP); differentiated colonies show lower staining intensity or are negative for AP activity. For the photospectrometric measurement of AP activity in mESCs, p-nitrophenol levels were determined using StemTAG Alkaline Phosphatase Activity Assay Kit (CBA-302; Cell Biolabs) following the manufacturer's protocol.

RNA expression analysis

For semi-quantitative PCR, total RNA from ES cells was purified using Qiazol lysis reagent (Qiagen). 1.5 µg of total RNA was treated with DNAse (RQ1, Promega) and subjected to reverse transcription in the presence of random primers (Promega) or oligo dT primers using SuperScriptIII Reverse Transcriptase (Invitrogen) according to the manufacturer's suggestions. The obtained cDNA was amplified by PCR on a 2720 Thermal Cycler PCR machine (Applied Biosystems) using AccuPrime Taq DNA Polymerase (Invitrogen) according to the manufacturer's instructions. PCR products were run on a 1.2% agarose gel and visualized by ethidium bromide staining. Specific PCR primers are shown in Supplementary table 1.

To detect antisense *Oct4* and *Oct4P4* transcripts, strand-specific reverse transcription polymerase chain reaction was performed. Gene-specific sense primers were used for reverse transcription; ***Oct4* asRNA: TCGATAGATGACACACAGGG; *Oct4P4* asRNA: GTCACGAATTCTCTCCGGGA), and gene-specific antisense primers were used to synthesize cDNAs to the sense RNA (Oct4 senseRNA: AGCTATCTACTGTGTGTCCC; Oct4P4 senseRNA: CAGTGCTTAAGAGAGGCCCT).** cDNA was synthesized using the Quantitect reverse transcription kit (Qiagen) according to the manufacturer's protocol. cDNA fragments were PCR amplified using a 2720 Thermal Cycler (Applied Biosystems) using AccuPrime Taq DNA Polymerase (Invitrogen) according to the manufacturer's instructions. PCR products were run on a 1.2% agarose gel and visualized by ethidium bromide staining. **Specific PCR primers used for the amplification of both sense and antisense Oct4 (Oct4 f; Oct4 r) and Oct4P4 (Oct4P4 f; Oct4P4 r) transcripts are reported in Supplementary table 1.**

For quantitative real-time PCR, total RNA was extracted from cells using Qiazol lysis reagent (Qiagen) according to manufacturer's suggestions. Nuclear and cytoplasmic RNA fractions were obtained by the following protocol: cells were collected, resuspended in

lysis buffer (10mM NaCl, 20mM MgCl, 10mM Tris-Cl, pH 7.8, 5mM DTT, 0.5% NP-40) and kept in ice for 5 minutes. Nuclei were pelleted by centrifugation at 8000 rpm for 5 minutes at 4°C, pellets were washed and resuspended in lysis buffer. The cytoplasmic fraction was collected to a new tube and clarified by centrifugation. Nuclear and cytoplasmic fractions were subjected to protease treatment for 20 minutes at 37°C by adding an equal volume of proteinase K solution (300 mM NaCl, 0.2 M Tris-Cl, pH7.5, 25mM EDTA, 2% SDS and 0.1 mg/ml proteinase K), and RNA was purified using the Qiazol lysis reagent (Qiagen).
The RNA was subjected to DNase treatment; cDNA was synthesized using the Quantitect reverse transcription kit (Qiagen) according to the manufacter's protocol. Quantitative real-time PCR was performed on StepOnePlus real-time PCR machine (Applied Biosystems), using SYBR Green Universal PCR Master Mix (Applied Biosystems). Oligonucleotides used for quantitative and semi-quantitative PCR are listed in Supplementary table 1.

Telomere repeat content measurements

Cells were collected, resuspended in proteinase K solution (50 mM Tris-Cl pH 8.0, 50 mM EDTA, 1% SDS, 10mM NaCl) and incubated o.n at 55°C adding 0.1 mg/ml Proteinase K (Invitrogen). After phenol/chloroform extraction and ethanol precipitation, total genomic DNA was resuspended in ddH_2O. To measure the teloemre repeat content, genomic DNA was diluted to 50 pg/ml and analyzed by RT-PCR on a StepOnePlus real-time PCR machine (Applied Biosystems), using SYBR Green Universal PCR Master Mix (Applied Biosystems) and specific telomere primers. A multicopy gene, Alu-equivalent B1 repeats, was used as reference gene for telomere length measurement.
The oligos used for quantitative RT-PCR are indicated below:
mB1: Forward: GCACCTTTAATCCCAGCAC; Reverse: TGAGACAGGGTTTCTCTGTA. Tel: Forward: CGGTTTGTTTGGGTTTGGGTTTGGGTTTGGGTTTGGGTT; Reverse: GGCTTGCCTTACCCTTACCCTTACCCTTACCCTTACCCT.

Supplementary Reference:

1. **Adamski, M. G., Gumann, P. & Baird, A. E. A method for quantitative analysis of standard and high-throughput qPCR expression data based on input sample quantity. *PLoS One* 9, e103917 (2014).**
